# Acoustic levitation and rotation of thin films and their application for room temperature protein crystallography

**DOI:** 10.1038/s41598-022-09167-z

**Published:** 2022-03-30

**Authors:** Michal. W. Kepa, Takashi Tomizaki, Yohei Sato, Dmitry Ozerov, Hiroshi Sekiguchi, Nobuhiro Yasuda, Koki Aoyama, Petr Skopintsev, Jörg Standfuss, Robert Cheng, Michael Hennig, Soichiro Tsujino

**Affiliations:** 1 Division of Biology and Chemistry, Paul Scherrer Institut, 5232 Villigen-PSI, Switzerland; 2 Photon Science Division, Paul Scherrer Institut, 5232 Villigen-PSI, Switzerland; 3 Nuclear Energy and Safety Research Division, Paul Scherrer Institut, 5232 Villigen-PSI, Switzerland; 4 Japan Synchrotron Radiation Research Institute, Kouto 1-1-1, Sayo-cho, Sayo-gun, Hyogo, 679-5198 Japan; 5 leadXpro AG, PARK InnovAARE, 5234 Villigen-PSI, Switzerland

**Keywords:** Acoustics, X-ray crystallography

## Abstract

Acoustic levitation has attracted attention in terms of chemical and biochemical analysis in combination with various analytical methods because of its unique container-less environment for samples that is not reliant on specific material characteristics. However, loading samples with very high viscosity is difficult. To expand the scope, we propose the use of polymer thin films as sample holders, whereby the sample is dispensed on a film that is subsequently loaded onto an acoustic levitator. When applied for protein crystallography experiments, rotation controllability and positional stability are important prerequisites. We therefore study the acoustic levitation and rotation of thin films with an aspect ratio (the diameter-to-thickness ratio) of 80–240, which is an order of magnitude larger than those reported previously. For films with empirically optimized shapes, we find that it is possible to control the rotation speed in the range of 1–4 rotations per second while maintaining a positional stability of 12 ± 5 µm. The acoustic radiation force acting on the films is found to be a factor of 26–30 higher than that for same-volume water droplets. We propose use cases of the developed films for protein crystallography experiments and demonstrate data collections for large single crystal samples at room temperature.

## Introduction

The airborne handling and manipulation of small particles and droplets via ultrasonic acoustic levitation have recently attracted attention in terms of chemical and biochemical analysis^1–16^. The combination of the containerless environment of acoustic levitation for small quantities (submicro- to a few microlitres) of samples^2^ with analytical tools such as optical spectroscopy^3^, Raman scattering^1,5–9,14^, X-ray and neutron diffraction^10,12^, and mass spectrometry^11,13,15^ has been demonstrated. In situ X-ray or neutron diffraction experiments with amorphous pharmaceuticals and biological macromolecules prepared by acoustic levitation have also been reported^17–20^. In the recently proposed acoustic levitation diffractometer (ALD)^21–23^, an ultrasonically acoustically levitated droplet containing protein crystals is irradiated by a highly brilliant X-ray beam, and X-ray diffraction images from all crystal orientations are collected by a high-frame-rate pixelated X-ray image detector, with a frame rate up to 3 kHz^22^. In this way, a dataset of a few thousand diffraction images can be acquired within a few seconds before the sample is damaged by the evaporation of the liquid contents. Sample rotation at less than a degree per frame acquired in a time of 0.3–1 ms with positional stability less than the sample size can achieve this with the photon fluxes available at state-of-the-art synchrotron beamlines^22^. However, experiments for highly viscous samples pose a challenge because of the difficulty of directly dispensing these samples onto the acoustic levitator. This is the case for protein crystal samples grown in *meso,* e.g., in the lipidic cubic phase (LCP)^24–29^. These types of samples include membrane proteins, for which structure determination by X-ray crystallography is very important for drug discovery^30,31^. For the purpose of widening the scope of the acoustic levitator application for the analysis of such samples, we explore the possibility of using an acoustically levitated thin film as a sample holder, wherein the samples are dispensed onto the film^32^.

However, experimental investigations on the acoustic levitation and rotation of thin films are rare^33^. Enhancement of the acoustic radiation pressure for a cylinder with an increasing aspect ratio of the sample, i.e., the ratio between the diameter and height, has been reported^34–36^ but only for an aspect ratio range below 10–20. When a nonspherical object is acoustically levitated, the coupling of the acoustic radiation pressure with the anisotropy of the object creates torque^37^, and the resultant fast rotation may cause positional instability and ejection from the levitator. Introducing asymmetry in the acoustic levitator by inserting a reflecting plate^38^ or creating an asymmetric acoustic field^39^ via holographic synthesis^40–45^ can lock the angular position and rotate the sample controllably but at the cost of the size of the instrument and increased demands for ultrasound drivers. The latter is especially the case when phased-array ultrasound transducers are used. Rotation of acoustically levitated objects by viscous torque has also been reported, whereby the angular momentum of the acoustic radiation is transferred to the levitated object through the air viscosity^46–48^. Nevertheless, the ultrasound acoustic pressure is required to be higher than 140 dB^46^, which limits practical applications.

Against this background, we study the characteristics of the acoustic levitation and rotation of thin films with an aspect ratio (diameter-to-thickness ratio) of 80–240 in a single-axis levitator as a function of acoustic pressure. Our results show that the acoustic radiation force is orders of magnitude larger on the thin film than on the spheres and that we can control the rotation up to tens of rotation per second (rps) with positional fluctuations on the order of 10 µm. Using these thin films as sample holders, X-ray diffraction experiments at room temperature are conducted for single protein crystal samples. We also propose a concept for experiments with small samples. The results of our pilot experiment show that the proposed method and the instrumentation are compatible with the room temperature experimental environment in synchrotron beamlines.

## Results

### Acoustic levitation and rotation of thin films

**Figure 1:**
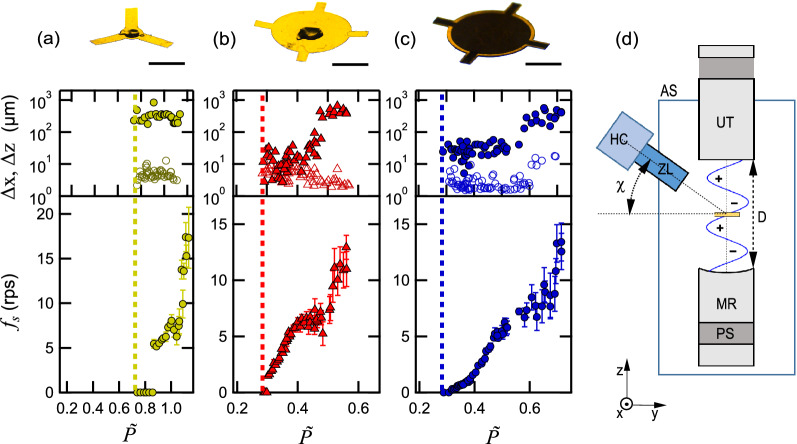
The relationships between the ultrasound pressure $\tilde{P }$ and the rotation rate *ƒ*_s_ and the positional stability ∆*x* (filled marks) and *∆z* (unfilled marks) in the radial and axial directions of the acoustically levitated thin films in a single-axis acoustic levitator excited at approximately 40 kHz. The results for three thin films T1, T2, and T3 in (**a**–**c**), with their shapes depicted by images taken from an oblique angle (*χ* = 30°) at the top of each panel, are shown. At the center of the thin films in (**a**) and (**b**), a small volume (subµL) of silicone grease is dispensed to emulate the conditions in protein crystallography experiments. In the case of (**c**), comprising a sandwich of two films similar to (**b**) but with an additional groove (dark area, see Supplementary Fig. S1), silicone grease is dispensed in the cavity between the films. $\tilde{P }$ is normalized by the threshold pressure to levitate a small (< 0.5 mm) water droplet in the same setup. The vertical dashed lines in (**a**–**c**) mark the threshold pressure for the levitation of each film. (**d**) The schematic experimental setup. *UT* ultrasonic transducer, *MR* mirror reflector, *PS* ultrasound pressure sensor, *HC* high-speed camera, *ZL* zoom lens, *AS* air shield. The UT-MR distance *D* was set approximately equal to 5*λ*/2, where *λ* is the acoustic wavelength in ambient air, and thin films were loaded at the middle (3rd from MR) node plane.

We studied the acoustic levitation and rotation of five thin film samples, T1-T5, with empirically optimized size and shapes in an acoustic levitator excited at 40 kHz ultrasound (see “Methods”). The films are fabricated from 25 µm- or 50 µm-thick polyimide foils (density of 1.9 g/cm^3^) with diameters comparable to one half of the acoustic wavelength. As shown in Fig. 1, T1 comprises three 3 mm-long blades, and T2 is a 4 mm-diameter disk with four short blades attached around the disk. T3 is a sandwich of two films with a shape similar to that of T2, but a groove with a depth of one-half thickness of the disk part is milled. T4 and T5 are also sandwich films fabricated from amorphous polyimide.

In Fig. 1, the variation in the rotation rate *ƒ*_s_ and the radial and axial positional stabilities Δx and Δz measured for a range of values of ultrasonic pressure $\tilde{P }$ in the acoustic cavity are shown for T1–T3. See Movies 1, 2, and 3 for videos of the three rotating samples recorded at a constant $\tilde{P }$. The setup is depicted in Fig. 1d. For T1 and T2, Fig. 1 shows cases where a small volume (subµL) of silicone grease (density of 1.2 g/cm^3^ and approximately the same as water and the LCP, within 10–20%) is dispensed at the middle (bottom side), which emulates dispensed LCP samples (see below). The results for unloaded T1 and T2 (see Supplementary Fig. S1) are similar to those of the loaded case. The characteristics of T4 and T5 are also similar to those of T3 (with some shift along $\tilde{P }$ for T5, see Supplementary Fig. S2). Samples with nominally the same shape (e.g., without a large bend) show approximately the same rotation characteristics (Supplementary Figs. S1, S2). In the case of T3, approximately 0.4 µL silicone grease is dispensed in the cavity between the two films. $\tilde{P }$ is measured by the pressure sensor attached to the mirror reflector. The vertical dashed lines in each panel indicate the threshold ultrasonic pressure ${\tilde{P }}_{th}$ necessary to levitate each sample.

There are two pressure regions: In the high $\tilde{P }$ region, the levitated thin films rotate as the rotation rate *ƒ*_s_ increases with increasing $\tilde{P }$, while in the low $\tilde{P }$ region, they do not achieve complete rotation. The pressure dividing the two regions is 3–10% larger than ${\tilde{P }}_{th}$, where the precision of the values depends on the film type and film flatness. The transition from the still to the rotating state is abrupt and characterized by a minimum rotation rate *ƒ*_s,min_. In the high $\tilde{P }$ region, *ƒ*_s_ can reach 15–20 rps with positional stability Δz in the vertical direction on the order of 10 µm. The horizontal positional stability Δx of T2 and T3 stays within tens of µm at low *ƒ*_s_ (below 7 rps), although Δx for T1 and for T2 and T3 at higher *ƒ*_s_ (> 7–15 rps) increases to above 100 µm.

The increase in Δx at high *ƒ*_s_ is found to be due to a shift of the rotation axis away from the axis of the levitator within the horizontal plane. However, the out-of-plane oscillation^33^ is very small in our thin films. Consequently, the precession of the rotation axis is negligible. This is particularly true for T2 and T3, with short side blades. We find that their presence is crucial for stabilizing the rotation. In fact, a disk-shaped thin film sandwich without short blades rotates along an axis lying in the disk plane. This effect, however, is absent for T3 over the same $\tilde{P }$ range, as demonstrated in Movie 4 ($\tilde{P }$ varying from 0.3 to 0.6 and then returning to 0.3). We note that for the protein crystallography experiments, the low *ƒ*_s_ region (1–4 rps and below) is the most suitable for acquiring diffraction images because of the overall sample positional stability and the fact that these rates are close to 0.1°–1° per acquisition time of 1 ms (see Methods), and the high *ƒ*_s_ region with increased Δx is not relevant.

**Figure 2:**
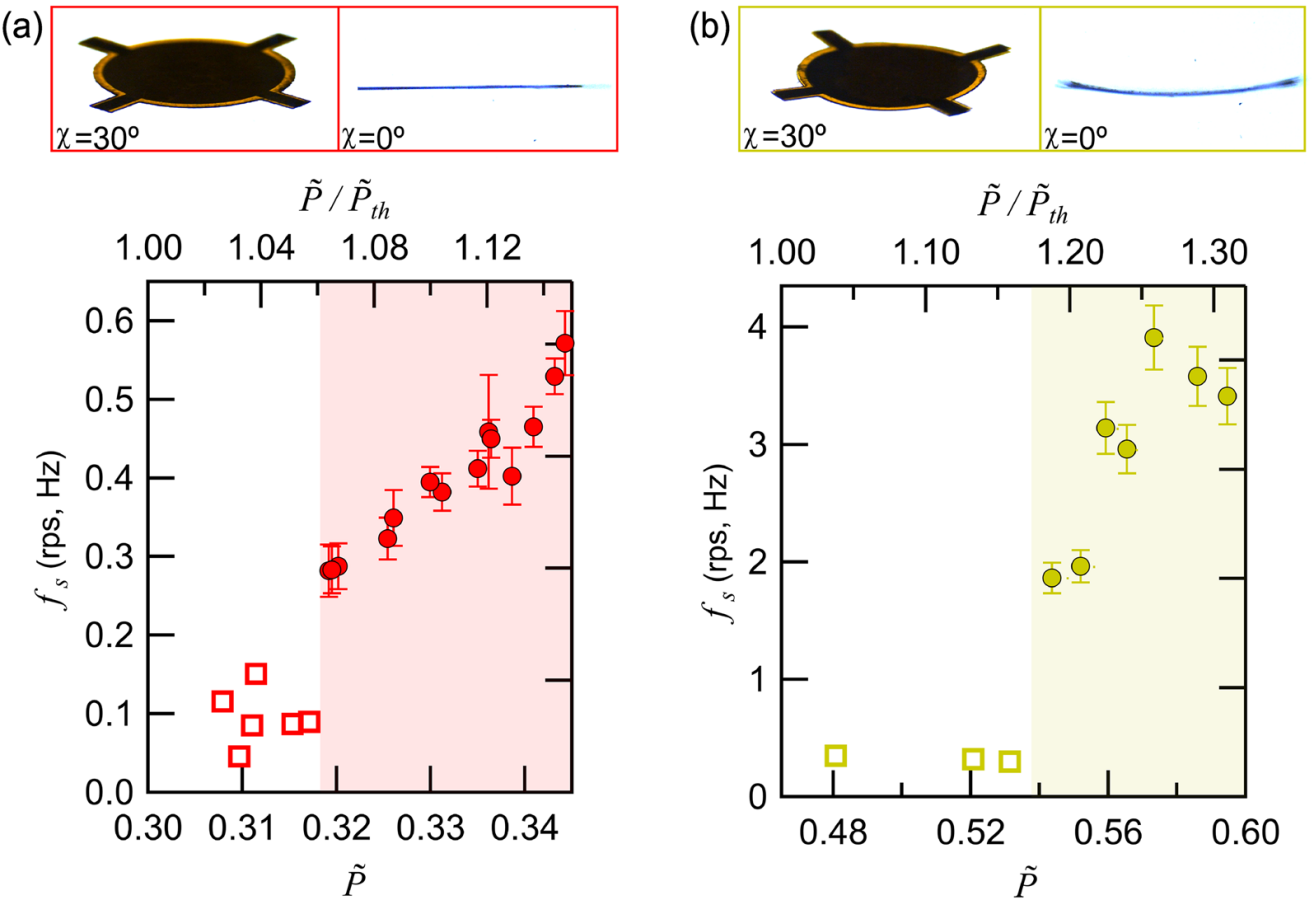
(**a**) The relationships between the ultrasound pressure $\tilde{P }$ and the rotation speed *ƒ*_s_ of T3 near zero rotation. When $\tilde{P }$ decreases from a high value (> 0.35) to the levitation threshold pressure ${\tilde{P }}_{th}$ (equal to 0.30), *ƒ*_s_ decreases monotonically (filled circles in shaded area) until a minimum rotation speed *ƒ*_s,min_ equal to 0.28 is reached at $\tilde{P }$ = 0.32. At lower $\tilde{P }$, angular oscillation within the horizontal plane with a frequency of approximately 0.1 Hz (unfilled squares) with random amplitude is observed. The pictures at the top show the levitated thin film from above (left, at 30°) and from the side (right, at 0°). (**b**) Same as (**a**) when the thin film sample is markedly bent with an overall bending angle of 19°. Compared with those of the flat sample in (**a**), higher *ƒ*_s,min_ and higher ${\tilde{P }}_{th}$ are observed. The top horizontal axes of the figures show $\tilde{P }$ divided by ${\tilde{P }}_{th}$ of the respective samples.

Close inspection of the levitated films in the low $\tilde{P }$ region shows that they display angular oscillation with frequencies of 0.05–0.18 Hz, as shown in Fig. 2, together with the rotation characteristics in the low *ƒ*_s_ region: Complete rotation occurs at *ƒ*_s_ above a minimum value of *ƒ*_s,min_ = 0.28 rps (filled circles) for $\tilde{P }$ larger than 0.32 (shaded area). As shown in Movie 5, the average angular position of the levitated thin film (T3) at $\tilde{P }$ = 0.3 returns to the same angle after an increase in $\tilde{P }$ to 0.5 and a decrease back to 0.3. The angular oscillation amplitude can reach 90° but varies randomly over time at a fixed $\tilde{P }$ and with changing $\tilde{P }$.

The finite value of *ƒ*_s.min_ indicates the presence of an angular locking potential within the horizontal plane. The locking of the angular position is compounded by the possible asymmetry of the sample. In the presence of the angular locking potential, full rotation requires that the kinetic energy of the rotation be higher than the potential height. This situation is similar to the case of a pendulum, wherein the oscillation amplitude and the kinetic energy need to be higher than the gravitational potential energy to switch from oscillating at the bottom to swinging around the pivot. We tentatively consider a small deviation in the acoustic field in the levitator from perfect cylindrical symmetry as the origin of the angular potential. For example, this might be caused by acoustic reflection from the setup surroundings. We note that intentionally breaking the cylindrical symmetry of a single-axis levitator by placing a small acoustic reflector nearby can lock the angular position of a nonspherical levitated object^38^. Furthermore, moving the reflector around the levitator rotates the levitated object (see Supplementary Fig. S3).

**Figure 3:**
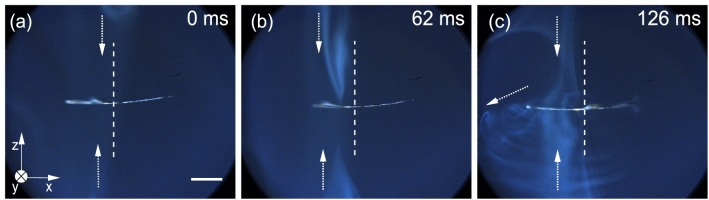
Airflow around the acoustically levitated and rotated thin film T1 visualized by incense smoke. The smoke is pulled along the levitator axis toward the thin film and pushed away once it reaches the thin film in the radial direction. The smoke fronts above and below the thin film were approximately 3 mm away from the thin film at 0 ms and were pulled toward the thin film along the levitator, as indicated by arrows (**a**); almost reached the thin film at 62 ms (**b**); and were pushed away in the radial direction by the edge of the rotating thin film with vertex shedding at the film edge (**c**). $\tilde{P }$ was equal to 1.05, and the rotation speed of the levitated thin film was 15 Hz.

The rotational characteristics are also affected by the deformation of the film. This influence is evidenced by the comparison of the T3 sample in Fig. 3a with negligible bending (overall bending below 0.7°) with the T3 sample in Fig. 3b with an overall bending of 19°. We observe *ƒ*_s,min_ to be an order of magnitude larger and the value of $\tilde{P }$ required to reach *ƒ*_s,min_ to be 70% higher for the latter case. Additionally, the angular oscillation frequency of the film at low $\tilde{P }$ is approximately twofold higher than that of the flat sample case. This observation shows the importance of acoustic scattering from the levitated film for the rotation characteristics. We also find that the acoustic radiation force is greatly modified in the levitation of the thin film samples, as described below.

The observed axial torque on the levitated thin films probably originates from the acoustic radiation pressure^49^ instead of other effects, such as viscous torque created by the circulation of the surrounding air^46,47,50,51^. This hypothesis is confirmed by visualizing airflow around the levitated and rotating thin film T1. See Fig. 3 and Movie 6, where the estimated rms ultrasound pressure *P*_ac_ is equal to 1.4 kPa (rms ultrasound velocity *V*_ac_ of 3.32 m/s) and ƒ_s_ = 15 rps: At first, air is directed toward T1 along the levitator axis from the upper and lower sides of T1 (Fig. 3a). When the air hits the film, it is pushed away from the film in the radial direction (Fig. 3b,c) while causing a vortex and shedding at the same time.

Since the acoustic radiation pressure is nominally cylindrically symmetric, torque around the levitator axis parallel to the direction of the acoustic standing wave is finite only when the acoustic radiation pressure by scattering from the thin film creates an asymmetry, e.g., when a part of the film is twisted (rotated around a radial direction) and the average twist over the film surface is finite, although this effect appears to be small. In the case of the T3 sample shown in Fig. 2a, we estimate that the twist is much smaller than 10^–3^ rad, although no precise measurement has been obtained yet. This is likely due to plastic deformation of the fabricated film. In fact, the direction of the rotation is normally unchanged with an increase or cyclic change in $\tilde{P }$. Furthermore, when a sample is loaded upside down, it rotates in the reverse direction with approximately the same rotation characteristics (see Supplementary Fig. S4), supporting our interpretation.

### Enhanced acoustic radiation pressure on levitated thin films

**Figure 4:**
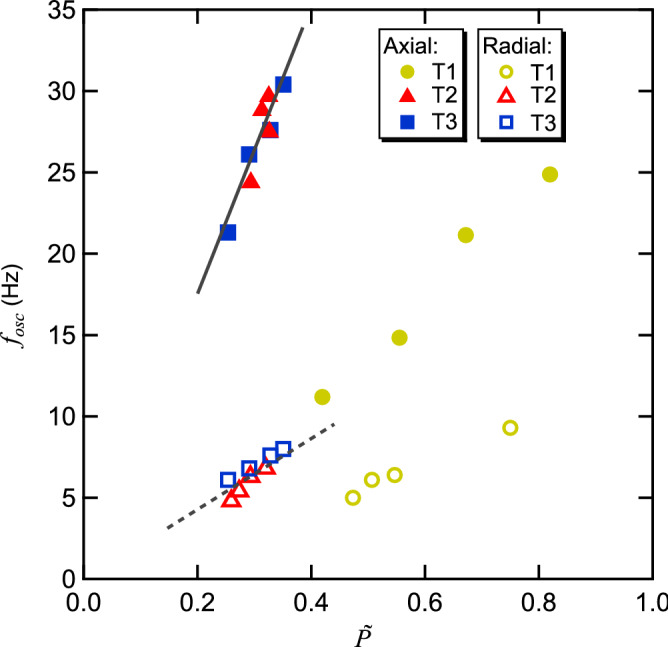
Resonance frequencies *ƒ*_osc_ of the axial (filled symbols) and radial (unfilled symbols) positional oscillations of the acoustically levitated thin films. *ƒ*_osc_ increases linearly with increasing $\tilde{P }$, similar to the water droplet case reported in Ref. 52 but at smaller slopes (indicated by the solid and dashed lines for the axial and radial oscillations, respectively, for the cases of samples 2 and 3) than the water droplets by a factor of 5.

Investigating the acoustic radiation force acting on the thin film samples, we note that the samples are acoustically levitated against gravity for acoustic pressures higher than ${\tilde{P }}_{th}$, wherein ${\tilde{P }}_{th}$ in the case of water droplets is equal to 1 through the normalization of the pressure. Therefore, the ${\tilde{P }}_{th}$ smaller than 1 for the thin films depicted in Fig. 1 shows that, at a given acoustic pressure, the acoustic radiation force on the films is higher than that on water droplets. To quantitatively study the acoustic radiation force, we measure the resonance amplitude oscillation frequencies *ƒ*_osc,z_ and *ƒ*_osc,x_ in the axial and radial directions, respectively^52^, and compare them with those of the water droplet case. The results are shown in Fig. 4. We find that the *ƒ*_osc_ values of T2 and T3 are approximately the same and change linearly with varying $\tilde{P }$. By comparing their slopes with those of water obtained in the same geometry in Ref.^52^, we find that the *ƒ*_osc,z_ and *ƒ*_osc,x_ of T2 and T3 are higher than those of the water case by factors of 5.1 ± 0.1 and 5.5 ± 0.2 at a given $\tilde{P }$, thereby enhancing the stiffness by 26.0 ± 1.0 and 30.3 ± 2.2, respectively. This observation is consistent with the literature^34–36^. We note, however, that these authors considered disks with aspect ratios that are 10–20 smaller than those in our case.

**Figure 5:**
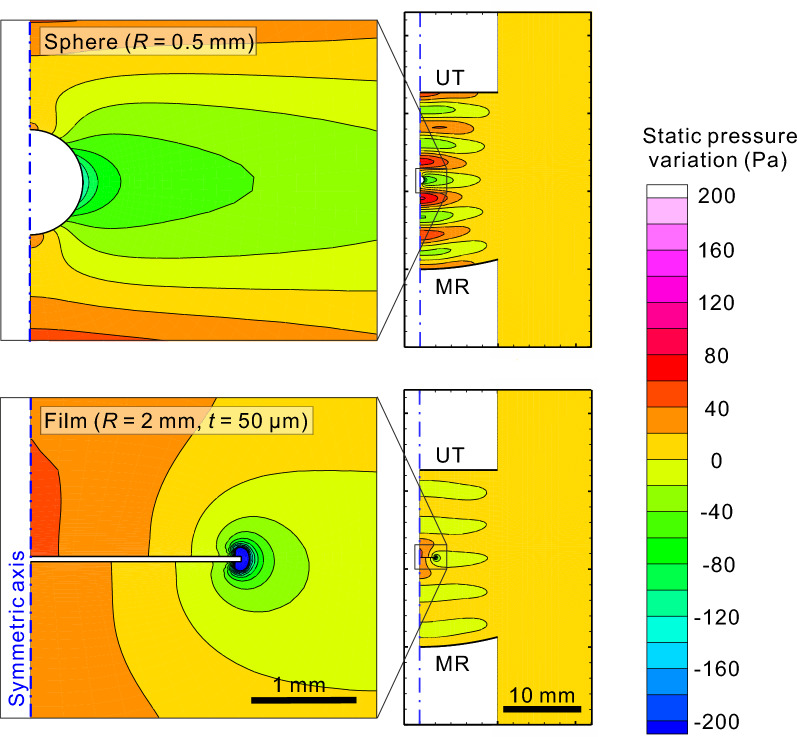
Time-averaged ultrasound-induced static pressure distribution in the single-axis acoustic levitator calculated by computational fluid dynamics simulation. This distribution was obtained as the difference between the total static pressure and the ambient pressure. The upper and lower panels show the case where a sphere (radius *R* = 0.5 mm) or a thin film disk (*R* = 2 mm and thickness *t* = 50 µm) was placed at the center of the middle node. The sphere and the disc were assumed to be rigid bodies (infinite Young’s modulus and acoustic impedance). The acoustic levitator was set at the 5th resonance in the absence of a sphere or film, and the edge of the UT vibrated at 40 kHz with an rms amplitude of 5 µm. *UT* ultrasound transducer, *MR* mirror reflector.

For comparison, we calculate the acoustic field of our single-axis levitator by using a computational fluid dynamics (CFD) simulation, in which the Navier–Stokes equations are numerically solved in the time domain (see “Methods”) in a two-dimensional axisymmetric coordinate system. The short blades attached to the peripheral part of the thin film sandwich are neglected because of the use of axisymmetric coordinates. The time-averaged pressure distributions for the sphere (0.5 mm radius) and the disk (2 mm radius and 50 µm thickness, with approximately the same volume as the sphere) are shown in Fig. 5 when they are placed at the middle-pressure node, i.e., velocity peak. As a consequence of the extensive acoustic scattering by the disk at the middle node, the stagnation of the acoustic velocity results in enhanced pressure on the disk surface. Figure 5 also shows that in comparison to the spherical case, when the disk is placed in the levitator, the pressure amplitude of the standing wave away from the disk is greatly decreased. A part of the pressure decrease is caused by the detuning of the resonance characteristics of the acoustic cavity of the levitator, but this is independent of the enhancement of the acoustic pressure on the levitated disk (see “Methods”).

Strong negative pressure is observed at the edge of the disk (see Fig. 5, bottom left), which we ascribe to the phase singularity of the acoustic wave induced by edge scattering^53^. To quantitatively evaluate the influence of edge scattering on the acoustic levitation force *F*_z_, the distribution and integral of the levitation force are calculated. The result shows that the influence of edge scattering on *F*_z_ is minor (below 10%, see Supplementary Fig. S6). Nevertheless, when the edge scattering is chiral, e.g., due to a small shape asymmetry of the sample, it may induce torque and rotate the disk. The detailed quantitative analysis of this torque mechanism in terms of the chiral bending of thin films requires 3-dimensional calculations and is beyond the scope of the present manuscript.

We also evaluate the slope of the $\tilde{P }$ dependence of the vertical harmonic oscillation frequency *f*_z_^(th)^. Here, *f*_z_^(th)^ is calculated from *F*_z_ at various elevations (see Method). The results are shown in Supplementary Fig. S7a, assuming a water density of 10^3^ kg/m^3^ for spheres and a density of 1.45 × 10^3^ kg/m^3^ (polyimide sandwich filled with grease) for disks. The results show that *f*_z_^(th)^ varies linearly with varying $\tilde{P }$ in both the spherical and disk cases and that the slopes *k*_*f*_^(s)^ of the *f*_z_^(th)^ − $\tilde{P }$ relationships of the small spheres are unchanged as the sphere radius is varied, as expected from theory^54^. However, the slopes *k*_f_^(d)^ of the *f*_z_^(th)^ − $\tilde{P }$ relationships of the disks increase proportionally with increasing disk diameter (see Supplementary Fig. S7b), indicating that the disk interferes extensively with the acoustic field. From the calculation, we find that the calculated ratio of the slopes between *k*_*f*_^(d)^ *and k*_*f*_^(s)^ agrees with the experimentally observed ratio of (5.1 ± 0.1) for a disk with a diameter equal to 2.5 ± 0.2 mm. This result is in adequate agreement with the size of the thin film T3 (note that the simulation neglects the short blades of T3).

### Application of thin films as sample holders for protein crystallography experiments

**Figure 6:**
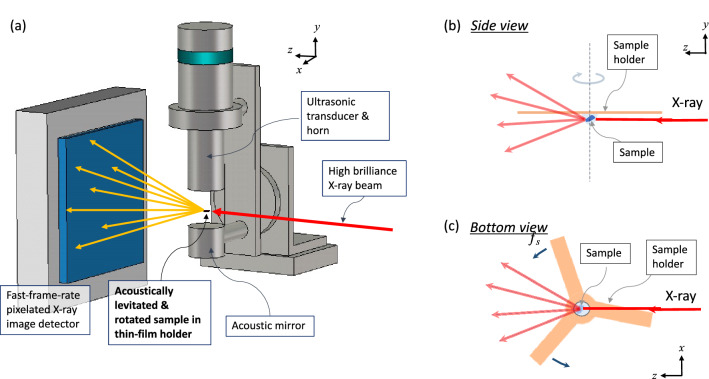
(**a**) Schematic setup of protein crystallography experiments using an acoustic levitation diffractometer (ALD) with a thin film sample holder for single-crystal samples. Samples are dispensed at the middle of the thin film sample holder and loaded into the acoustic levitator. The sample rotates together with the rotation of the thin film sample holder within the horizontal plane. When the X-ray beam irradiates the sample, X-ray diffraction images of the sample from all crystal orientations are continuously recorded by the fast-frame-rate pixelated image detector, as schematically shown in the side view (**b**) and bottom view (**c**).

**Figure 7:**
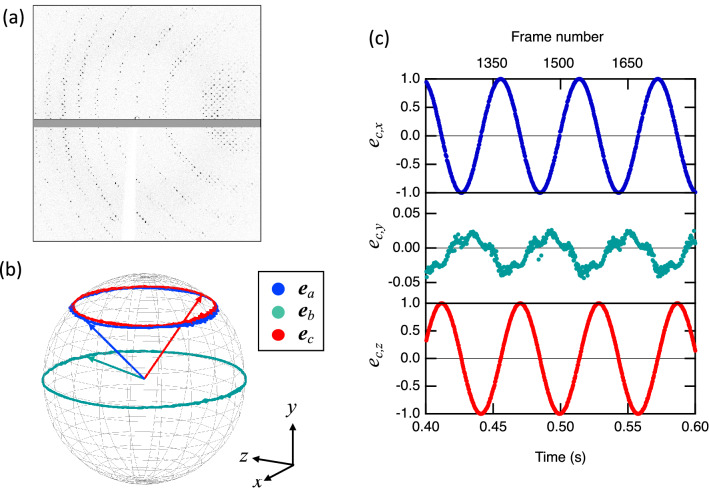
(**a**) A diffraction image of a single-crystal lysozyme sample with a size of 200 µm collected by ALD using the thin film sample holder T1. This is the 25th of 3000 images acquired at a 3 kHz frame rate for 1 s with a nearly 100% hit rate. (**b**) The trajectory of the normalized lattice vectors ***e***_a_, ***e***_b_, and ***e***_c_, showing the evolution of the crystal lattice vectors ***a***, ***b***, and ***c*** during the data collection. The vectors show the lattice orientation at time zero. All the trajectories rotate around the y-axis of the detector frame (vertical direction), as expected from the rotation of the levitated sample holder, with the axis along the levitator axis in the vertical direction. The X-ray beam irradiates the sample parallel to the *z*-axis from the negative to the positive direction. (**c**) The *x*-, *y-*, and *z*-components of the vector ***e***_c_ for the period of 0.4 to 0.6 s. The small e_*c*,y_ shows that the c-axis of the crystal is approximately parallel to the thin film holder.

Room temperature X-ray diffraction experiments of protein single crystals were proposed and conducted at the ALD installation on the beamline X06SA at the Swiss Light Source (SLS) in the Paul Scherrer Institut, Switzerland. Fig. 6 shows the schematic setup. In Fig. 7, we show the results for a lysozyme crystal with a size of 200 µm in the LCP dispensed on a T1 holder. At a rotation rate of 17 rps and a detector frame rate of 3 kHz, a nearly 100% hit rate and index rate (the fraction of the diffraction images successfully indexed) are reached. In Fig. 7b, we show the evolution of the lattice orientations as the sample rotates during 1 s of data collection: ***e***_a_, ***e***_b_, and ***e***_c_ are the unit vectors along the lattice directions ***a***, ***b***, ***c***, respectively. Arrows indicate the lattice orientations at time zero. The observation that the trajectories of ***e***_a_, ***e***_b_, and ***e***_c_ are circles parallel to the *x*–*z* plane with approximately constant *y*-components (see the coordinate system definition of the experiment in Figs. 6a and 7b, wherein the X-ray beam is along the *z*-axis) agrees with the fact that the rotation axis of the levitated thin film is along the levitator axis in the vertical orientation (*y*-direction). The relative orientation between the crystals and film is generally random (in particular, when several small crystals are dispensed on the film). However, in the experiment depicted in Fig. 7 with the large sample, ***e***_b_ moves approximately along the equator, indicating that one of the crystal facets is parallel to the film. The small modulation of the oscillation of the *y*-component of ***e***_c_ in Fig. 7c demonstrates that the diffraction measurement sensitively detects the residual precession of the rotating film (less than 1%), which is difficult to detect by high-speed camera recordings.

**Figure 8:**
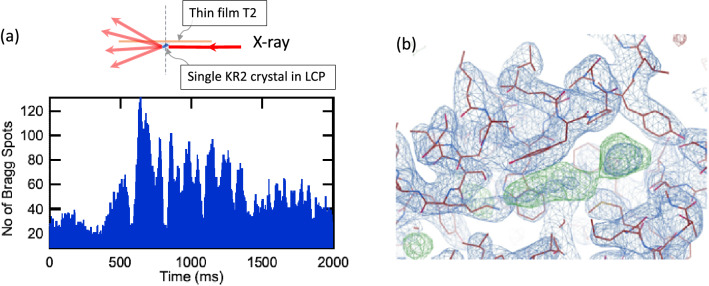
Results of the ALD diffraction experiments for KR2 single-crystal samples grown in LCP using a T2 thin film holder. The sample was dispensed at the middle of T2. A low ultrasound pressure setting was used, wherein a slow angular excursion of the sample of approximately 90° was performed during the data collection with a duration of 2 s, corresponding to an oscillation frequency in the range of 0.1 Hz (see Fig. 3a). With a detector frame rate of 1 kHz, 2000 diffraction images recording 20–130 Bragg spots are shown in the bottom panel of (**a**). Data processing successfully indexed 75% of these images, and the crystal structure of the KR2 sample was successfully determined at a resolution of 2.5 Å (CC_1/2_ equal to 0.1). (**b**) The electron density of the determined structure.

We next applied the method to the light-driven sodium ion pump *Krokinobacter eikastus* rhodopsin 2 (KR2) grown in the LCP in a microsyringe, which is a membrane protein sample with a smaller size and weaker diffraction intensity than those of the lysozyme sample shown above. The sample size is in the range of (80–150) × (50–90) µm^2^ with a thickness of 10 µm (see^55^ and “Methods”). The LCP is extruded from the syringe under inspection with a microscope. When a crystal is found in the LCP, we dispense it onto the film with a volume below 0.5 µL. For this experiment, we use T2, which has higher positional stability than T1 and is advantageous for small samples. We also set the levitator to a low ultrasound pressure range, wherein the thin film exhibits an angular oscillation with a frequency of 0.1 Hz (see Fig. 3a). When the oscillation range is on the order of 90° with a rotation angle per frame equal to 0.05°, nearly all 2000 diffraction images of the collected dataset contain more than 20 Bragg spots (Fig. 8a). The processing of this dataset exhibits an index rate of 76% with a completeness close to 80% at a nearly geometry-limited resolution of 2.5 Å given by the highest resolution shell with a CC_1/2_ of 0.1^56^. Merging two additional datasets (a total of 6000 images with an index rate of 77%) results in 100% completeness at the same resolution (see Supplementary Table S2). The high index rate shows the positional stability of the levitated sample. Achieving high completeness for high-resolution shells is a results of the rotation control of the levitated thin film sample holder: with the high positional stability of the rotating sample holder, the X-ray beam irradiates the crystals throughout the exposure. By integrating the datasets, we determine the crystal structure of the sample by the molecular replacement method, as shown in Fig. 8b (see Supplementary Material for the statistics of the datasets).

## Discussion

Our experimental investigation supports the ability to control the rotation of acoustically levitated polymer thin films with optimized shapes for slow rotation rates of 1–4 rps with a positional stability of 12 ± 5 µm. As demonstrated in the pilot experiments, this condition is compatible with room temperature X-ray diffraction experiments. With the acquisition of datasets within a few seconds, the sample damage from dehydration during data collection is mitigated. In contrast to acoustic rotation via viscous torque in liquid^47^ with a much higher viscosity than that in air, acoustic torque on levitated polymer thin films is produced by coupling acoustic radiation pressure with small asymmetry in the films. This result is evidenced by the visualization of airflow around the rotating thin film in the acoustic levitator. Nye and Berry noted that acoustic waves scattered in air from rough surfaces and edges contain dislocations or phase singularities^53^, which can carry angular momentum, such as screw dislocations and acoustical vortices^58,59^. In fact, although not chiral, our CFD simulation in Fig. 5 shows a singularity at the film edge. In contrast, a small chirality in the thin film samples, e.g., caused by imperfections from the fabrication process, would produce torque under scattering of the incident acoustic wave by the surface roughness and edges of the thin films. We note that despite the subwavelength dimensions of the thin films, the effect can be finite. This result agrees with the sensitivity of the rotation characteristics to the flatness of the thin films observed in Fig. 2a. The precise quantitative understanding of the acoustic torque observed in our experiments will be a subject of future research.

Our study shows the enhanced stiffness and force of acoustic radiation on levitated thin films. In comparison to same-volume water droplets, the enhancement is 26–30-fold for the levitated films with aspect ratios larger than 100. The CFD simulation reproduces the observations well. By inspecting the simulation results, we observe that the acoustic pressure on the film is increased by the large stagnation of the acoustic velocity due to the insertion of films into the pressure node (or velocity peak). We note, however, that even though the acoustic radiation pressure acting on the levitated thin film surface is enhanced, the expected transmission of ultrasound inside the film is small. It is known that transmission through a large acoustic impedance medium can become negligible with a small thickness^60^. In the case of the polyimide film (density 1.9 × 10^3^ kg/m^3^) used here, this thickness limit is approximately 100 nm. This suggests that the samples inside the thin film sandwich, as well as in the droplet on the film, are immune to the high acoustic pressure of the levitator and the damage it may cause^21^.

In comparison to phased-array-transducer levitators^39–41^, our setup using a single-axis acoustic levitator is simple, compact, and robust. Exploiting these properties, we integrated the acoustic levitator into the protein crystallography beamline of a synchrotron facility and conducted room temperature crystallography experiments on LCP samples. For single protein crystals with sizes of 80–100 µm, we show that the loaded crystals can be irradiated by an X-ray beam throughout the data collection. This enables the collection of Bragg reflections from all around the reciprocal space with a minimal number of samples within a short time before the dehydration of the sample.

**Figure 9:**
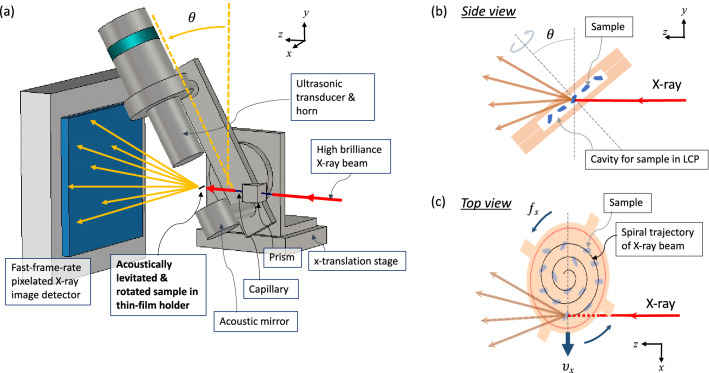
(**a**) Concept of the serial protein crystallography experiments using the acoustic levitation diffractometer (ALD) and the thin film sample holder for microcrystalline LCP samples. As shown in (**b**) and (**c**), the samples are dispensed within the cavity formed inside the thin film sandwich, which is subsequently loaded into the acoustic levitator with its axis tilted from the vertical direction by *θ* for irradiation from the X-ray beam at the top of the thin film sandwich at an oblique angle. While the levitated sample rotates, the levitator is mechanically translated at a speed *v*_x_. In this way, the X-ray beam spot traces a spiral trajectory, as shown in (**c**). The pitch of the neighboring trajectory and the beam irradiation area during the shutter time is controlled to maximize the sample efficiency and SNR by adjusting the rotation speed and the translation speed.

Further extension of the present method for microcrystal experiments is under study. For this, we consider the use of film sandwich sample holders (such as T3), wherein crystals in the LCP are dispensed within the cavity of two films. As shown in Fig. 9, the film sandwich is levitated with an acoustic levitator with its axis tilted by 30° from the vertical direction. While the sample is levitated and rotated at a constant speed, it is moved in the transverse direction at a constant velocity at the same time. In this way, the X-ray beam on the sample holder traces a spiral trajectory, for which the pitch is determined by the rotation speed and the translation velocity. We find that similar rotation control with positional stability can be achieved in such a tilted acoustic levitator configuration (see Supplementary Figs. S10–S11). The demonstrated positional stability of 12 ± 5 µm indicates that the crystals with a size of 10–30 µm dispersed in the film sandwich can be uniformly illuminated by the X-ray beam by adjusting the rotation speed, the translation speed, and the beam spot size. A potential advantage of such a method is the fast data collection time: a scan over a 4 mm-diameter area with a 10 µm-beam-spot-size can be completed within 8 s. We tested the concept of the spiral-scan data collection using the ALD with the film sandwiches at the BL40XU beamline at SPring-8 with a high flux beam (with an effective energy width of 5.5%, see “Methods” and Supplementary Figs. S9, S12–S15). However, further optimization of the experiment is required to improve the data quality of the dataset, and the processing of datasets collected with such a large bandwidth beam is beyond the capabilities of the current state-of-the-art software. These are the subjects of the present intense research and will be reported elsewhere.

Two of the representative state-of-the-art methods for microcrystal experiments are the viscous jet method^61^ and the fixed-target method^62,63^. In comparison, similar to the fixed target method, the proposed spiral-scan method using ALD is expected to attain higher sample efficiency than the viscous jet method. Although the polymer film produces background scattering to reduce the signal-to-noise ratio, the total thickness of 50 µm for T3 and T4 is the same as the typical diameter of a 50 µm viscous jet^61^. In our method, we expect further reduction of the background with the use of thinner films. We also find that amorphous polyimide film exhibits no structured background, unlike Kapton film (see the Supplementary Material, in particular, the diffraction images of Supplementary Figs. S12–S14 using Kapton film and that in Supplementary Fig. S15 with the amorphous polyimide film). This is advantageous for attaining a higher signal-to-noise ratio. We also note that in the reported experiments of the fixed-target method, the scan area is limited to a few hundred microns, and the scan time is longer in the 2D raster scan^62,63^ in comparison to the spiral-scan method due to the overhead of turnaround at edges. Nevertheless, for the efficient measurement of the samples in the range of a few microns using the sandwich method, further technological development of the acoustic levitator with improved positional stability will be crucial.

## Methods

### Single-axis ultrasound acoustic levitator

We used a single-axis acoustic levitator comprising a bolt-clamped Langevin-type lead-zirconate-titanate transducer attached to an aluminum horn with a diameter of 20 mm and a concave mirror reflector (diameter and focal length of 20 mm). We drove the transducer at the resonance frequency, approximately equal to 39 kHz, and adjusted the acoustic cavity to the *n*-th resonance, with the distance *D* between the horn and the mirror approximately equal to *nλ*/2, where *λ* is approximately equal to 8.8 mm in ambient air at 20 °C, nominally with *n* = 5 or 7. The excitation power for the acoustic levitation of thin films ranged from 0.5 to 1.5 W. The resonance of the acoustic cavity was monitored by the pressure sensor fixed to the mirror reflector. Because of the use of the focusing reflecting mirror, the ultrasound pressure *P*_ac_ and the corresponding ultrasound velocity *V*_ac_ = *P*_ac_/(*rc*) around the 3rd node from the mirror reflector were approximately twofold larger than the ultrasound pressure on the mirror (*r* = 1.2445 kg/m^3^ and is the density of air, and *c* = 343 m/s and is the speed of sound in ambient air). For a small (0.5 mm or smaller diameter) levitated water droplet, the proportional factor between the sensor signal and *P*_ac_ acting on the levitated droplet at the 3rd node of the 5th resonance levitator was evaluated by identifying the threshold pressure to levitate the droplet, which was equal to 1.35 kPa according to analytical theory^54^ considering the acoustic field distribution of our levitator^52^. The reported $\tilde{P }$ value was normalized with respect to the threshold value of 1.35 kPa for levitating a small water droplet (diameter smaller than 0.5 mm) in the same setup, as reported in Ref.^52^.

### Measurement of the levitation and rotation characteristics of thin films

At a given ultrasound pressure, the rotation or angular oscillation characteristics, as well as the positional stability, were evaluated from video recordings from the side or at an oblique angle using a FASTCAM mini AX100 high-speed camera (Photron Inc.) with a zoom lens attached. The frame rate of the recording was typically adjusted between 125–6000 fps, and the shutter time was adjusted to 1–10 ms. To avoid possible air disturbance, the acoustic levitator setup was normally wrapped by a transparent plastic sheet.

### Thin film sample holders

Samples T1, T2, and T3 shown in Fig. 1a–c were laser-cut from 25 µm-thick preformed polyimide foils. T1 consisted of three 3 mm-long and 0.5 mm-wide symmetrically arranged blades, T2 was a 4 mm-diameter disk with four 1 mm-long and 0.5 mm-wide blades, and T3 was a sandwich of two films with similar dimensions to T2, except that each film constituting T3 was thinned to 12 µm within the central 3.9-mm diameter area (dark area in Fig. 1) by laser ablation. Similar to T3, Samples T4 and T5 were also sandwich thin films; however, T5 was twice as thick as T3 and T4. T4 and T5 were fabricated from photolithography and cross-linking from photosensitive polyimide resin. In the case of T5, the thickness of single film was equal to 50 µm (the depth of the groove was equal to 25 µm). The polymer thin films (T3) also exhibited background molecular structures (see Figs. S12, S13, and S14). This was why we tested T5, which was fabricated from photosensitive polyimide, from which we expected shorter cross-linked polymers with more randomized orientations than T3, which was fabricated from a preformed polyimide film. The comparison between the diffraction images in Fig. S15 measured with T5 and those in Supplementary Figs. S12–S14 measured with T3 showed that the structured background ascribed to the fibrous structure of the polyimide observed in T3 was absent in T5. The total thickness of the thin film sandwich, including the liquid part, was of the same order of magnitude as the diameter of the jet produced by the viscous jet extruder^61^.

### CFD simulation

The commercial CFD code ANSYS® Fluent Version 2020 was used for the flow analysis. The governing equations were mass conservation equations, momentum equations, energy equations, and the equation of state of the ideal gas law. These equations were discretized in space with the finite-volume method. A second-order upwind scheme was used for the special discretization of the convection terms, and a second-order central scheme was used for the diffusion terms. A second-order accuracy implicit scheme was used for the time discretization. In the simulation, we modeled the object as a rigid body (with infinite acoustic impedance) since the acoustic impedance of water or polymer is a factor 10^3^ higher than that of air. A two-dimensional axisymmetric coordinate system was used under the assumption of the axial symmetry of the single-axis levitator. The short blades attached to the periphery of T2–T5 were difficult to model in the axisymmetric coordinate system and therefore were neglected for simplicity. To evaluate the force acting on the objects, we calculated the acoustic field when the objects were placed at several fixed vertical positions instead of solving the equation of motion, which largely simplified and shortened the simulation. The transducer oscillation was set to 40 kHz with an amplitude of 5 µm. The atmospheric pressure value was subtracted to highlight the acoustic radiation pressure. Time averaging was performed for four periods of acoustic oscillation after a constant pressure amplitude was attained.

The ultrasound transducer was modeled as an oscillating wall, where a sinusoidal motion was given as a function of time. The pressure wave could be generated owing to the oscillation wall, and the reflection waves from the oscillation wall could be considered because of the Neumann boundary condition applied to the pressure on the wall. The boundary condition applied to the far field of the computational domain was a pressure outlet boundary with a nonreflecting acoustic wave model. Together with the wall boundary applied to the mirror reflector, standing pressure waves were generated between the transducer and the reflector.

The levitating object, i.e., a water droplet or a thin film, was modeled as a rigid-solid sphere and disk, respectively. The position of the levitating object was fixed in space, and the motion of the levitating object was not determined because of our computational resource limitations. Instead of solving the equation of motion, several cases of simulations with different elevations of the object were computed, and the acoustic radiation force was calculated. The elevation of the object was ± 0.5 mm from the middle-pressure node, and the variation in the elevation was sufficiently smaller than the acoustic wavelength (8.7 mm). The harmonic oscillation frequency *ƒ*_z_^(th)^ of the levitated object in the vertical direction was estimated from the computed acoustic radiation force in the z-direction *F*_z_ as *ƒ*_z_^(th)^ = (-{(∂*F*_z_/∂*z*)/*m*}^1/2^/2π, where *m* is the mass of the levitating object. The dimensions of the levitating object were as follows: three sphere cases with radii of 0.3, 0.5 and 0.7 mm and five disk cases with radii ranging from 0.5 mm to 2.5 mm at an increment of 0.5 mm. The thickness of the disk was 50 μm for all cases. The densities of the sphere and the disk were set to 10^3^ kg/m and 1.45 × 10^3^ kg/m^3^, respectively, to calculate *m*.

The computational mesh was generated using the software Pointwise® Gridgen. A multiblock structured mesh was employed since it is more appropriate than an unstructured mesh for calculating the propagation of the pressure waves. The total number of mesh cells was 7452. Thin meshes were used around the levitating object to resolve the velocity boundary layer; the mesh thickness adjacent to the object was one micrometer. The dynamic mesh technique implemented in Fluent was used to incorporate the oscillation of the ultrasound transducer.

First, mesh-size and time-increment dependency studies were performed for the case without a levitating object. Convergence was obtained for the acoustic radiation force acting on the mirror reflector when (i) the mesh size in the wave propagation direction was smaller than 1/32 of the wavelength and (ii) the time increment was smaller than 1/40 of the wavelength. When the frequency of the transducer was 40 kHz, the mesh size had to be be smaller than 0.27 mm, and the time increment had to be 0.625 μs. Second, validation of the CFD simulation was performed for the case without a levitating object. The computed acoustic radiation force acting on the mirror reflector agreed with the measurement as a function of the distance between the transducer and the reflector.

After the mesh and time dependency studies and the validation, simulations of the levitating object were performed. The transient simulation was performed until a periodic condition had been attained. This condition was typically attained after 100 pressure waves, and the simulations were continued until 254 waves. The time-averaged acoustic radiation force acting on the levitating object was calculated during the last four waves.

### Influence of the disk-loading-induced detuning of the acoustic cavity on the enhancement of the acoustic pressure on the disk

As described in the main text, a part of the pressure decrease was caused by the detuning of the resonance characteristic of the acoustic cavity of the levitator, but this was independent of the enhancement of the acoustic pressure on the levitated disk: Loading a thin film sample into the levitator indeed shifts the resonance distance *D* between the transducer and the mirror reflector by 10% (see Supplementary Fig. S5); loading the sample in the levitator at a *D* that is resonant in the empty cavity leads to a decrease in $\tilde{P }$ by approximately a factor of 2. However, since the $\tilde{P }$ measured at the pressure sensor attached to the mirror reflector is proportional to the acoustic standing wave amplitude, the CFD simulation (Fig. 5) confirms that the detuning does not influence the relationship between *ƒ*_osc_ and $\tilde{P }$, especially the observed enhancement of the acoustic pressure on the thin film surface.

### Protein crystal preparation

Large lysozyme crystals were formed by the vapor diffusion method. Five microliters of protein solution (50 mM hen egg-white lysozyme) was mixed with 5 µL of crystallization buffer (125 mM NaCl, 100 mM sodium acetate, pH 4.65). Crystals were grown for 5–7 days to the appropriate size with 500 µL of reservoir in the well.

The microcrystalline lysozyme crystals were grown by the following procedure. Lysozyme solution (Sigma–Aldrich Ref: L2879, 100 mM sodium acetate pH 3.0, 25 mg/mL) and precipitant (100 mM sodium acetate pH 3.0, 28% NaCl, 8% PEG 6 K) were mixed in an Eppendorf tube and gently inverted 8–10 times. Crystals were grown overnight at 20 °C.

Crystallization of KR2 crystal samples in the lipidic cubic phase (LCP) was performed using conditions similar to those described in^55^ and^64^. Purified protein buffer (100 mg/mL KR2, 50 mM Tris pH 8.0, 150 mM NaCl, 0.05% DDM, 0.01% CHS, see^55^ for purification protocol) and monoolein (1-oleoyl-rac-glycerol, Nu-Chek prep) were thoroughly mixed in a 4:7 v/v ratio through coupled gas-tight Hamilton syringes. The formed LCP was extruded through Hamilton needles into plastic B-Braun Omnifix-F syringes loaded with precipitant (200 mM sodium acetate pH 4.4, 150 mM MgCl_2_, 35% PEG 200). Crystallization occurred overnight in the dark at 20 °C and yielded plate-like blue KR2 crystals with dimensions of (10–30) × (10–25) × (1–3) μm^3^. Thicker (~ 10 µm) and larger (80–150) × (50–90) µm^2^ KR2 crystals were grown by applying the same procedure, except the pH of the sodium acetate buffer was set to 4.6, and the crystals grew for five days.

A_2_A microcrystals in the LCP were grown inside a Hamilton syringe as described in Ref.^57^. Briefly, a purified A_2_A receptor at 25–30 mg/mL was mixed with 90% monoolein/10% cholesterol (w:w) at a protein:lipid ratio of 2:3 (w:w). Crystallization was initiated inside a Hamilton syringe by injecting the protein:lipid mixture into a mother liquor solution (0.1 M sodium citrate pH 5.0, 0.05 M sodium thiocyanate, 28–34% PEG 400, 5 mM ZM241385, 2% (v/v) 2,5-methylpentanediol) at a 1:10 (v:v) ratio. Crystals grown to their maximum size within 2 weeks were harvested by injecting the mother liquor solution into another Hamilton syringe. The swollen LCP was then topped up with extra monoolein in a stepwise manner to absorb excess precipitant and to transform the LCP back into the more viscous/less hydrated cubic phase. The sample was then ready for data collection.

### X-ray diffraction experiments

Experiments on single-crystal ALD diffraction experiments with the scheme depicted in Fig. 6 were conducted in the X06SA beamline at the SLS synchrotron facility, Switzerland, using the ALD installation in the beamline^22,23^. A pixelated high frame rate X-ray image detector, an EIGER X 1 M (Dectris AG), was used to detect the diffraction images, and a high-speed camera, a FASTCAM mini AX100, was used to monitor the acoustically levitated samples. An optical prism was inserted in the beam path to observe the sample with the high-speed camera via a zoom lens (a similar setup is depicted in Supplementary Fig. S8). Using the XYZ stage supporting the ALD, the position of the levitated crystal was aligned with the X-ray beam. The ultrasound pressure of the levitator, the alignment of the acoustic cavity of the levitator and the sample position alignment were controlled from a PC and could be adjusted from outside of the beamline hatch.

For the lysozyme crystal experiment shown in Fig. 7, an X-ray beam with a photon energy of 12.4 keV and a beam spot size of 42 µm × 32 µm (H × V, FWHM) was used. With a rotation rate of 17 rps and a detector frame rate of 3 kHz, the rotation angle per frame was approximately equal to 2°. The sample-to-detector distance was set equal to 100 mm. The resolution of the data was equal to 2.1 Å (see Supplementary Table S1), which was limited by the geometry (the highest resolution given at the corners of the diffraction image in Fig. 7a); see Supplementary Table S1.

For the KR2 experiment shown in Fig. 8, an X-ray beam with a photon energy of 10 keV (the photon flux is the highest at this photon energy, approximately equal to 10^12^ photons/s) was used. To reduce the parasitic dose for the radiation-sensitive LCP samples and to minimize the background scattering (to maximize the SNR), we used a narrow beam with a size of 42 µm × 25 µm (HxV, FWHM). The distance between the sample and the detector was set to 90 mm, which yields a geometry-limited resolution at the side of the detector of 2.3 Å. Diffraction images were collected with a detector frame rate of 1 kHz at an exposure time of 2 s (2000 images per dataset). The resolution of the data was 2.5 Å, given by CC_1/2_ equal to 0.1 (see Supplementary Table S2).

### Data processing of the X-ray diffraction datasets

The acquired X-ray diffraction datasets from large crystals in the film sample holders were processed with CrystFEL^65^ ver. 0.8.0. Xgandalf^66^ was used as an indexer in indexamajig. The partiality of the datasets was estimated by partialator using the xsphere calculation model. The electron density of KR2 shown in Fig. 8 was calculated by the molecular replacement method with CCP4 software^67^. We processed the datasets collected at BL40XU at SPring-8 by CrystFEL ver. 0.9.0. with the developer version of pinkindexer^68^.

## Supplementary Information

Supplementary Information 1.

Supplementary Information 2.

Supplementary Movie 1.

Supplementary Movie 2.

Supplementary Movie 3.

Supplementary Movie 4.

Supplementary Movie 5.

Supplementary Movie 6.

## Data Availability

All the data are available upon request.
