# Publisher Correction: Acoustic levitation and rotation of thin films and their application for room temperature protein crystallography

**DOI:** 10.1038/s41598-022-11111-0

**Published:** 2022-04-25

**Authors:** Michal. W. Kepa, Takashi Tomizaki, Yohei Sato, Dmitry Ozerov, Hiroshi Sekiguchi, Nobuhiro Yasuda, Koki Aoyama, Petr Skopintsev, Jörg Standfuss, Robert Cheng, Michael Hennig, Soichiro Tsujino

**Affiliations:** 1 Division of Biology and Chemistry, Paul Scherrer Institut, 5232 Villigen-PSI, Switzerland; 2 Photon Science Division, Paul Scherrer Institut, 5232 Villigen-PSI, Switzerland; 3 Nuclear Energy and Safety Research Division, Paul Scherrer Institut, 5232 Villigen-PSI, Switzerland; 4 Japan Synchrotron Radiation Research Institute, Kouto 1-1-1, Sayo-cho, Sayo-gun, Hyogo, 679-5198 Japan; 5 leadXpro AG, PARK InnovAARE, 5234 Villigen-PSI, Switzerland

Correction to: *Scientific Reports* 10.1038/s41598-022-09167-z, published online 30 March 2022

The Supplementary Information files published with this Article contained errors. The Supplementary Movies 2, 3, 4, 5 and 6 were incorrectly given. Supplementary Movie 2 was a duplicate of Supplementary Movie 1. As a result, Supplementary Movies 2, 3, 4 and 5 were incorrectly given as Supplementary Movies 3, 4, 5 and 6 respectively. Furthermore, the correct Supplementary Movie 6 was omitted.

These errors have now been corrected in the Supplementary Information section that accompanies the original Article.

