# Supplementary material for: Acoustic levitation and rotation of thin films and their application for room temperature protein crystallography
Source: Sci Rep. 2022 Mar 30;12:5349. doi: 10.1038/s41598-022-09167-z (PMC8967846; doi:10.1038/s41598-022-09167-z)
Supplement: Supplementary file 1 — Supplementary Information 1. [file 41598_2022_9167_MOESM1_ESM.pdf]

Supplementary Materials

Acoustic levitation and rotation of thin films and their application for room temperature protein crystallography

Michal. W. Kepa[1], Takashi Tomizaki[2, *], Yohei Sato[3], Dmitry Ozerov[2], Hiroshi Sekiguchi[4], Nobuhiro Yasuda[4], Koki Aoyama[4], Petr Skopintsev[1], Jörg Standfuss[1], Robert Cheng[5], Michael Hennig[5] & Soichiro Tsujino[1, **]

[1] *Division of Biology and Chemistry, Paul Scherrer Institut, 5232 Villigen-PSI, Switzerland*

[2] *Photon Science Division, Paul Scherrer Institut, 5232 Villigen-PSI, Switzerland*

[3] *Nuclear Energy and Safety Research Division, Paul Scherrer Institut, 5232 Villigen-PSI, Switzerland*

[4] *Japan Synchrotron Radiation Research Institute, Kouto 1-1-1, Sayo-cho, Sayo-gun, 679-5198 Hyogo, Japan*

[5] *leadXpro AG, PARK InnovAARE, CH-5234 Villigen-PSI, Switzerland*

* Corresponding author: takashi.tomizaki@psi.ch
** Corresponding author: soichiro.tsujino@psi.ch

Description on the X-ray diffraction experiments and beamline optics at BL40XU, SPring-8

Description on the evaluation of the average sample spacing from the observed hit rate for the X-ray diffraction experiments using the sandwich sample holder for dataset shown in Supplementary Figure S15

Supplementary Figure S1. Comparison of rotation characteristics of different thin film sample holders for T1, T2, and T3.

Supplementary Figure S2. Comparison of rotation characteristics of different thin film sample holders for T4 and T5.

Supplementary Figure S3. Lock and drag of the angular orientation of the levitated thin film sample (T3) by a small reflector.

Supplementary Figure S4. Comparison of rotation characteristics of a T3 sample when the sample is flipped up-side down.

Supplementary Figure S5. The relationship between the normalized pressure and the distance between the transducer and the mirror reflector.

Supplementary Figure S6. Partial integration analysis of F_z calculated by CFD.

Supplementary Figure S7. Calculated vertical harmonic oscillation frequencies of acoustically levitated sphere and disc, and their ultrasound pressure dependences.

Supplementary Figure S8: Schematic optics of the high photon flux beam experiment on BL40XU beamline at SPring-8 and the influence of capillary on the background scattering.

Supplementary Figure S9: Spectrum of the high photon flux beam at the BL40XU beamline at SPring-8.

Supplementary Figure S10: Rotation characteristics of T3 in the 30°-tilted levitator than in the vertical levitator.

Supplementary Figure S11. Rotation characteristics of T4 and T5 in the 30°-tilted levitator than in the vertical levitator.

Supplementary Figure S12: Picture of lysozyme microcrystals and their X-ray diffraction image measured by ALD with the large bandwidth and high photon flux beam measured by S3.

Supplementary Figure S13: Picture of A_2A microcrystals and their X-ray diffraction image measured by ALD with the large bandwidth and high photon flux beam measured by S3.

Supplementary Figure S14: X-ray diffraction image of KR2 microcrystals measured by ALD with the large bandwidth and high photon flux beam measured by S3.

Supplementary Figure S15: Snapshots of T5 during the spiral scan X-ray diffraction experiments and X-ray diffraction image of KR2 microcrystals measured by ALD with the large bandwidth and high photon flux beam measured by T5.

Supplementary Table S1: Statistics of the dataset of single-crystal lysozyme sample measured by ALD.

Supplementary Table S2 Statistics of the dataset of single-crystal KR2 samples measured by ALD.

X-ray diffraction experiments and beamline optics at BL40XU, SPring-8

Serial crystallography experiments for microcrystals using acoustically levitated thin film sandwich holders were conducted on a BL40XU beamline at the SPring-8 synchrotron facility, Japan, with a photon energy of 15 keV. The bandwidth of the beam in full width at half maximum is equal to 1%. However, because of the highly asymmetric spectrum shape with the pronounced low energy tail (see Supplementary Figure S10), 84% of the integrated photon flux (corresponding to the region within ± 1 standard deviation in the case of a Gaussian shape spectrum) spreads within the fraction of the energy range equal to 5.5%, which we defined as the effective width of the spectrum in the main text. The X-ray beam spot size σ and the photon flux measured at the sample position were equal to 13 ± 2 µm-rms and 2×10^{13} photons/s, respectively. The ALD was transported from the setup at the X06SA beamline at SLS. A gonio stage was attached to the XYZ stage of the levitator to tilt the levitator axis from the vertical orientation. A pixelated high frame rate X-ray image detector, EIGER X 1 M (Dectris AG), was used to detect the diffraction images, and a FASTCAM mini AX200 high-speed camera (Photron Inc.) was used to monitor the acoustically levitated sample holders.

In the experiment depicted in Figure 11, the photon flux and the beam size (FWHM) at the sample position were equal to 10^{13} photons/s and 13.7×15.3 µm^2 (horizontal × vertical), respectively. To minimize the background scattering by ambient air and to match the beam size with the crystal size from 10-30 µm, we inserted a pinhole and a capillary, as shown in Supplementary Figure S9. A tantalum pinhole with a diameter of 10 µm is placed at the beam waist. After the pinhole, the X-ray beam passed through the ion chamber for online flux monitoring. Subsequently, the collimated beam passed through a tantalum capillary with a diameter of 1 mm and a silver capillary with a diameter of 0.5 mm. The capillary ended at positions approximately 12 mm from the samples. The distance was chosen to avoid possible interference by the capillary on the acoustic standing wave in the levitator. The distance from the pinhole to the sample was 100 mm, and the X-ray beam spot size at the sample position was measured by a knife-edge scan. A prism was placed between the pinhole and the sample to monitor the optical image of the levitated specimen during the diffraction experiment by a high-speed camera. The capillaries were fixed on the prism. The experiments depicted in Supplementary Figures S10 and S10 were conducted in optics without the optimized setup (without capillary) with a 20-µm-diameter pinhole with approximately the same photon flux and beam size at the sample position (FWHM) approximately equal to 50×50 µm^2.

We set f_s = 3-4 rps and v_x = 0.25 mm/s and acquired diffraction images at a 1 kHz frame rate for 8 s. During data collection, the 26 µm-wide (in 2σ) X-ray beam spot irradiates microcrystals while tracing along the spiral trajectory with a transverse period of 62 µm, as determined by v_x/f_s. This narrow spacing led to the high sample efficiency, as discussed in the main text (see Supplementary Figures S10 and S11 for the crystal images and diffraction images of other samples using S3). No visual damage on the thin films was observed. We collected 15 datasets from 15 disk sandwiches in total, each of which consisted of 8000 diffraction images acquired from a film sandwich loaded with 0.3-0.4 µL-LCP (0.7 µL in the case when T5 was used). Therefore, a total volume of 5-10 µL of the LCP sample was used.

Evaluation of the average sample spacing from the observed hit rate for the X-ray diffraction experiments using the sandwich sample holder for dataset shown in Supplementary Figure S15

Assuming the density of microcrystals ρ and the values f_s (= 4 rps) and v_x (= 0.25 mm/s), the exposure time per frame τ (= 1 ms), the radius of the film R (=2 mm), the probability of hitting a sample P_n in the n-th frame is equal to $2\pi r_n \tau v_x \rho$, where r_n is given by $(R - v_x n \tau)$ $(0 \leq n \leq N)$ with $N = R/(v_x \tau)$ equal to 8×10^3. Hence, the hit rate given by the average probability $P = \langle P_n \rangle$ to hit a sample in each frame over n is given by $R v_x t\, r$. Equating this expression with the observed hit rate of 6.2%, we obtain r = 39.5 mm^2. As described in the main text, the ideal hit rate for samples with a density of 56±1 mm^{-2} is equal to 8.8±0.2%, wherein the sample density was estimated from the dispensed crystals with a size larger than approximately 20 µm (see Fig. 11 (b)). The density is equal to 73±8 mm^{-2} for crystals larger than approximately 10 µm, and the evaluated ideal hit rate is equal to 11.4±1.2%.

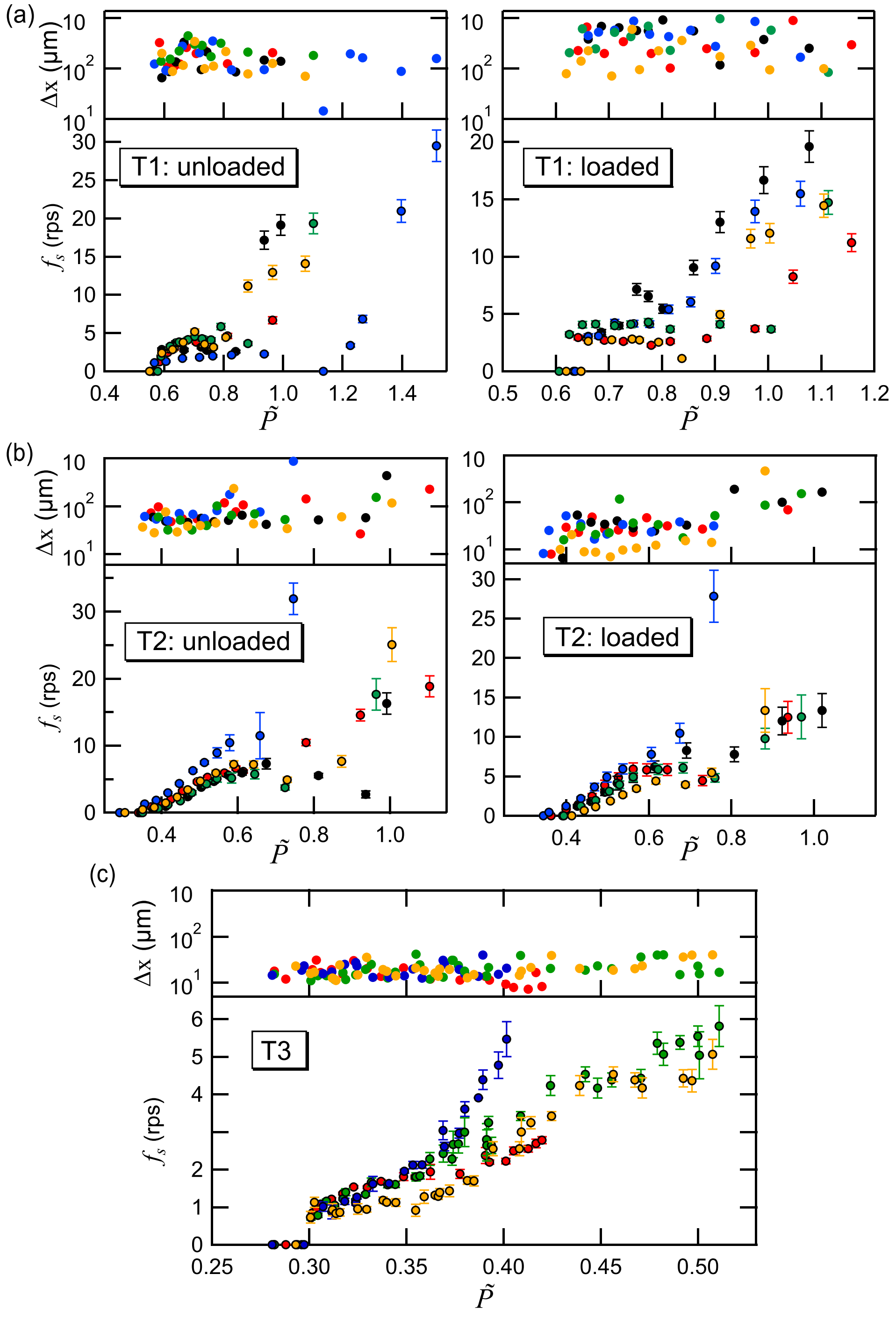

Supplementary Figure S1. Comparison of rotation characteristics of different thin film samples. (a) Five different T1 without (left) and with (right) dispensed grease. (b) Five different T2 without (left) and with (right) dispensed grease. (c) Four different T3. T1 and T2 display nominally same rotation characteristics at the rotation speed f_s below 5 rps, both in loaded and unloaded condition. The four T3 samples also show the same rotation characteristics. The small variation of the rotation speed of T3 samples, that is partly ascribed to the non-uniform loading and distribution of the silicone grease.

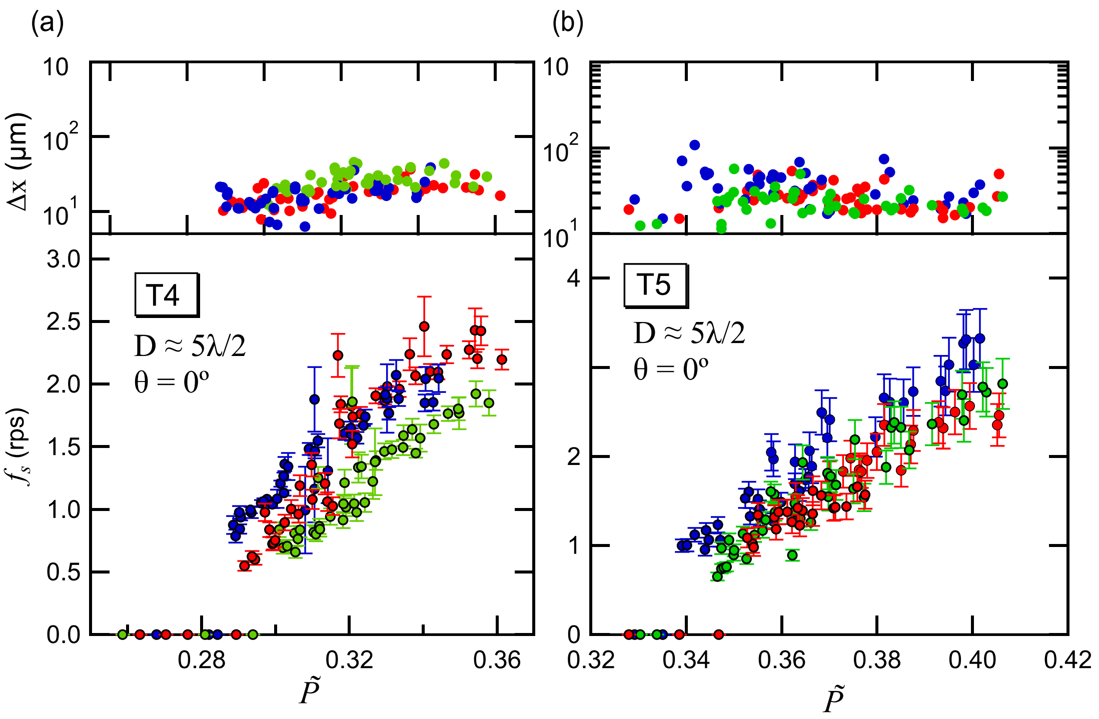

Supplementary Figure S2. Rotation characteristics of thin film sandwich samples T4 and T5 fabricated from photo-sensitive polyimide. The dimension of T4 in (a) is same as T3: the thickness of the single-film is 25 μm , wherein the middle part has recess with the depth of 12.5 μm. The dimension of the disc part and the leg part of T5 in (b) is same as T3 but the thickness of the single-film is 50 μm with the thickness of the middle part equal to 25 μm. The rotation characteristics of T4-type thin film sandwich samples are approximately same as that of T3, see Fig. 1 (c) and Supplementary Figure S1 (c). The rotation characteristics of T5-type samples is also similar to those of T3 and T4, except the levitation threshold pressure and the pressure of the minimum rotation speed are 10-20% higher. The higher levitation threshold pressure of S5 is ascribed to its mass to be twice heavier than T3 and T4.

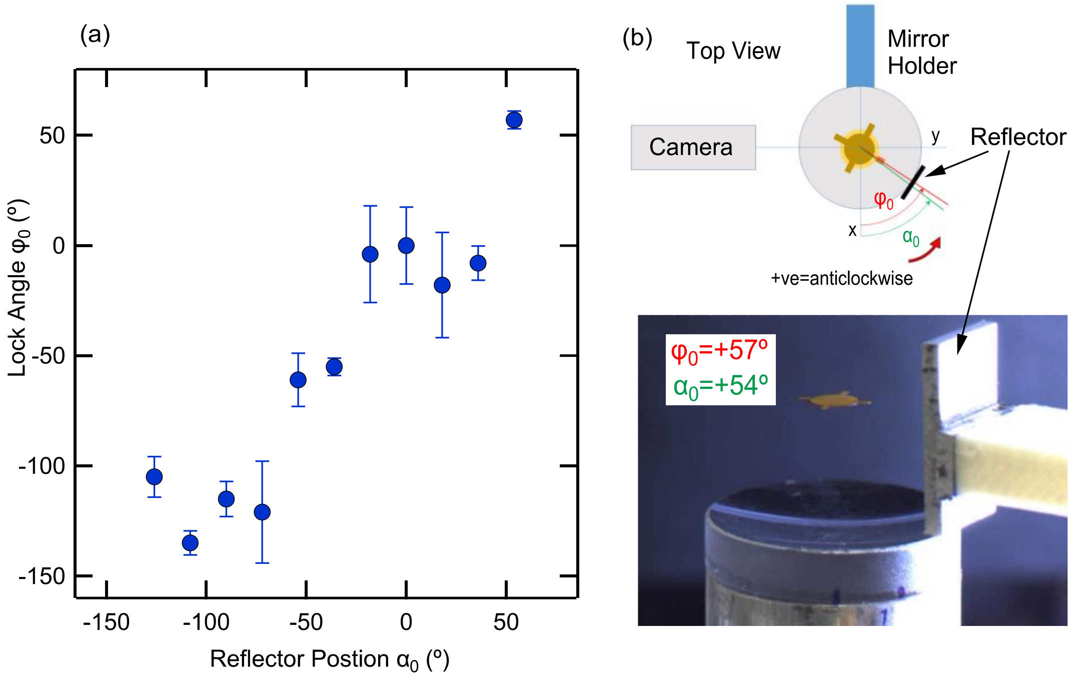

Supplementary Figure S3: Locking and rotation of angular position of a levitated thin film (T3) by a narrow acoustic reflector inserted at the side of the acoustic levitator. The lock angle φ_0 of the levitated thin film is dragged by rotating the reflector angle α_0. φ_0 and α_0 are defined in the schematic of the right top panel. The step structure of the φ_0 - α_0 relationship shows the slip-stick motion, suggesting the interference of the existing asymmetry of the levitator and the modulation of the acoustic field by the reflector plate. The picture (right bottom) shows the case when the thin film was locked at $\varphi_0 = 57°$ by $\alpha_0 = 54°$. The ultrasound pressure $\tilde{P}$ was equal to 0.35. In the absence of the side reflector, the levitated thin film rotates at the speed equal to 0.6 rps at this $\tilde{P}$.

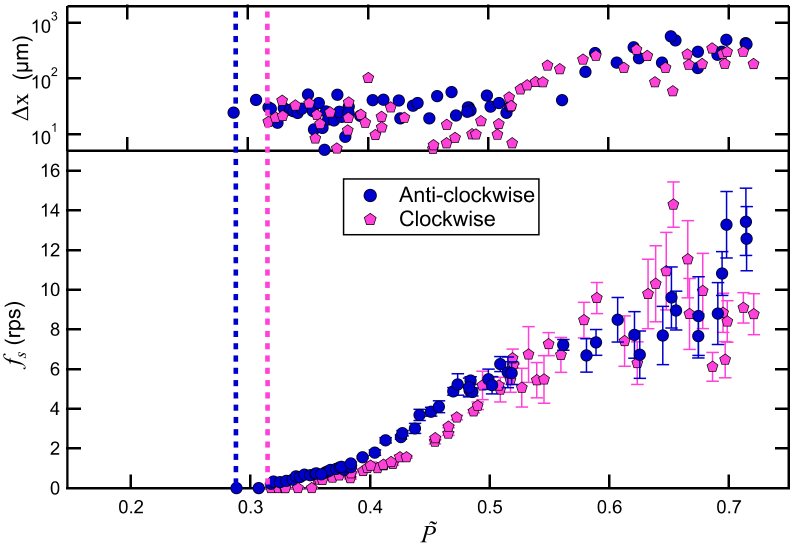

Supplementary Figure S4. Rotation characteristics of a T3 sample: Flipping upside down reverses the rotating direction with approximately the same rotation characteristics. The vertical dotted line indicates the approximately same levitation threshold values of the both cases.

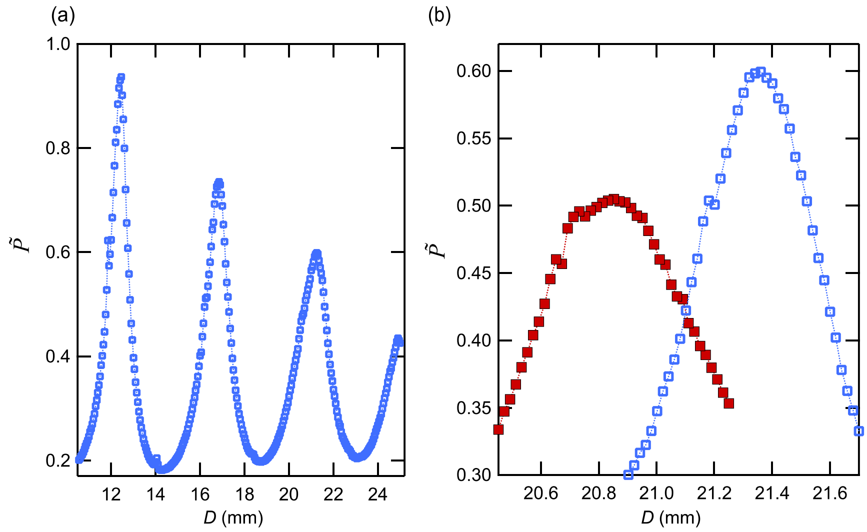

Supplementary Figure S5. The relationship between the normalized pressure $\tilde{P}$ and the distance D between the transducer and the mirror reflector. Note that $\tilde{P}$ is measured by the pressure sensor attached to the mirror reflector. (a) the resonance characteristics of the 3rd, 4th, and 5th resonances in the absence of sample. (b) The resonance characteristic when a thin film sample T3 is loaded (red filled squares) of the 5th resonance. The blue empty squares show the case without the sample shown in (a). The transducer excitation voltage was set constant during the measurement of (a) and (b) with and without the thin film sample.

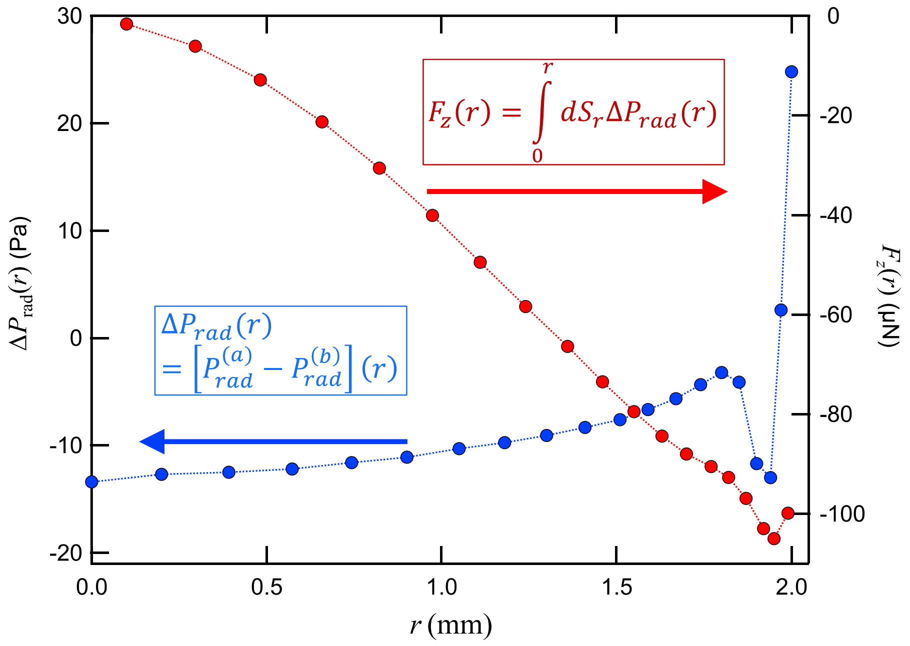

Supplementary Figure S6. Partial integration analysis of F_z showing that the contribution of the pressure singularity at the edge of disc is less than 12%. The difference $\Delta P_{rad}(r)$ of the acoustic radiation pressure P_{rad} (as obtained from the time-averaged pressure in Fig. 5) at the radial position r is defined as the difference of P_{rad} above the disc $P_{rad}^{(a)}$ from Prad below the disc $P_{rad}^{(b)}$. Note that these are calculated when the disc is placed at the center of the middle node, and the net acoustic radiation force F_z is downwards. The disc position where F_z counters gravity is lower that the center of the middle node by a few millimeters, of which exact value is a function of the ultrasonic transducer excitation and the pressure amplitude of the acoustic standing wave.

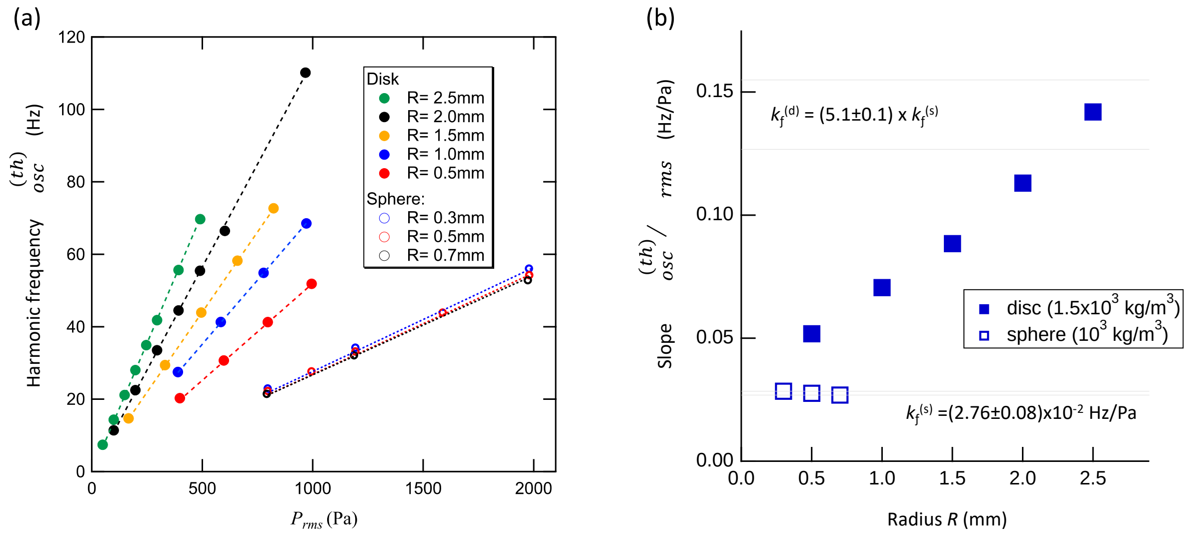

Supplementary Figure S7. (a) The relationship between the vertical harmonic oscillation frequency of the acoustically levitated sphere and disc and the ultrasound pressure P_{rms}. Same as the experiment, the average ultrasound pressure amplitude at the surface of the mirror reflector defines P_{rms}. The calculated assumes perfectly rigid body: a sphere with the radius of 0.3, 0.5, and 0.7 mm, or a disc with the diameter between 0.5 and 2.5 mm with the thickness of 50 μm. For comparison with experiments for small water droplets (density of 10^3 kg/m^3) or for thin film sandwich filled with silicon grease (average density of 1.45x10^3 kg/m^3), $f_{osc}^{(th)}$ was evaluated from the calculated acoustic radiation force F_z from the relationship, $-dF_z/dz = m\,(2\pi\ f_{osc}^{(th)})^2\ z$, with the mass m calculated from the volume and the density of respective objects. (b) The dependence of the slope of the $f_{osc}^{(th)}$-P_{rms} relationships calculated in (a) and the radius R of the levitated objects.

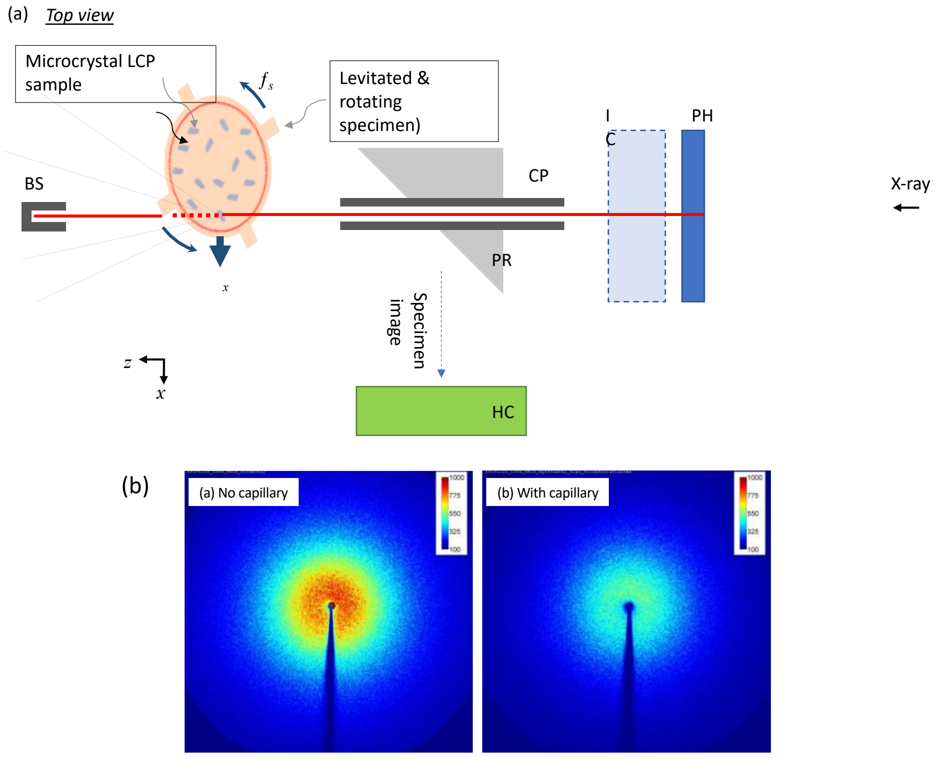

Supplementary Figure S8. (a) Schematic of the optimized beam optics for the high-photon-flux ALD experiment at BL40XU beamline at SPring-8. PH = pin-hole, IC = ion chamber, CP = capillary, PR = prism, HC = high-speed camera, BS = beam stop. (b) The effectiveness of the reduction of the background air scattering by the insertion of the capillary is demonstrated by comparing the bream distribution on the detector with (left) and without (right) capillary.

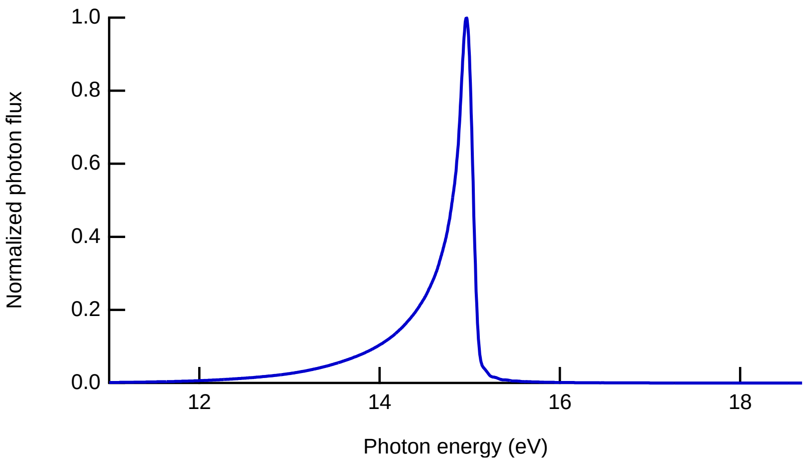

Supplementary Figure S9. Spectrum of the X-ray beam at BL40XU beamline at SPring-8. The peak position is equal to 14.96 keV and the full-width at the half-maximum (FWHM) is equal to 235.1 eV. FWHM is 1.57% of the peak photon energy (1.03% in the lower energy side and 0.54% in the higher energy side). Due to the large low energy tail of the spectrum, 60.2% of the integrated spectrum (within ±1 standard deviation in the case of a Gaussian shape spectrum) spreads between 14.14 and 14.96 eV (shaded area in the figure), equal to 5.5% of the peak photon energy.

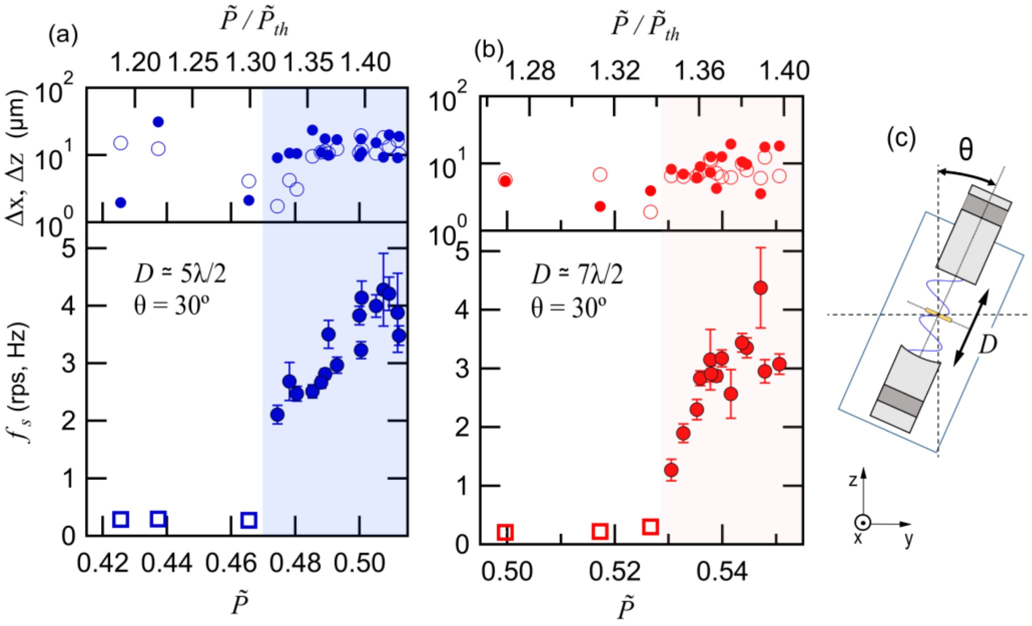

Supplementary Figure S10: The variation in the rotation speed of T3 with the variation in the ultrasound pressure when the single-axis acoustic levitator is tilted by $\theta = 30°$ from the vertical direction. (a) and (b) show the observed results in the case when the acoustic cavity is adjusted to the 5th and 7th resonance, respectively. In both cases, thin film sandwich S3 is loaded to the 3rd node from the mirror reflector. The top panels show the positional stability of the thin film sandwich in the axial (Δz, empty circles) and radial (Δx, filled circles) directions.

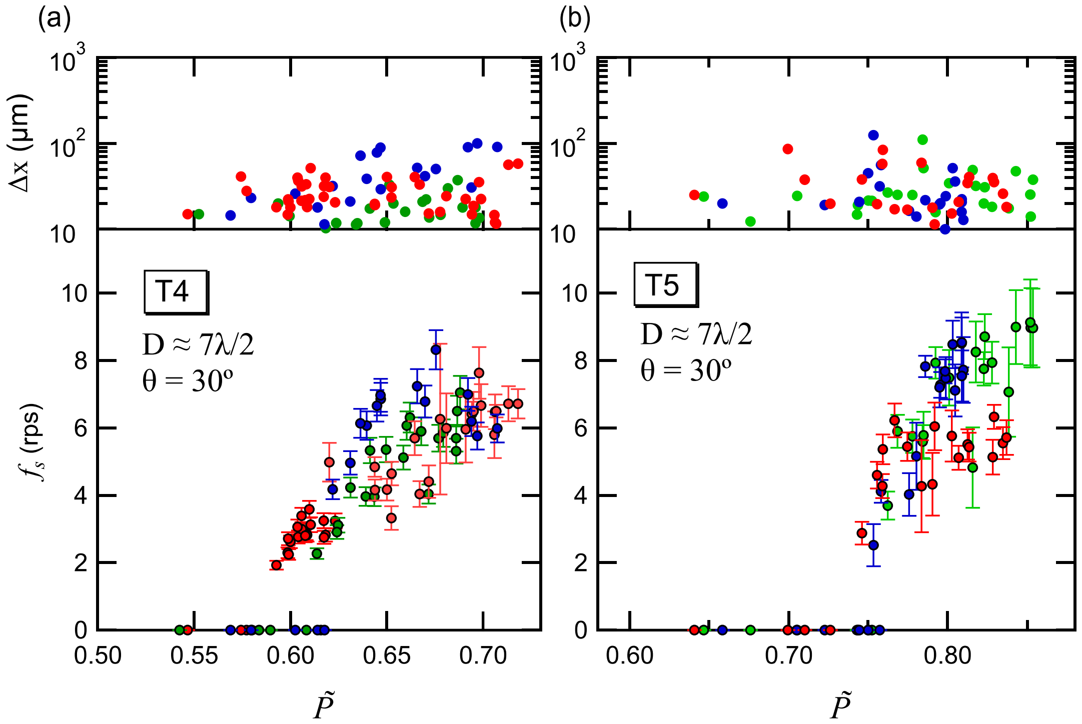

Supplementary Figure S11: Rotation characteristics of T4 (a) and T5 (b) levitated in the 7th resonance with the levitator axis tilted by 30° from the vertical orientation. As in the case of T3-type thin film sandwich samples, the minimum-oscillation-speed pressure values of both T4 and T5 are higher in the 30°-tilted levitator than in the vertical levitator.

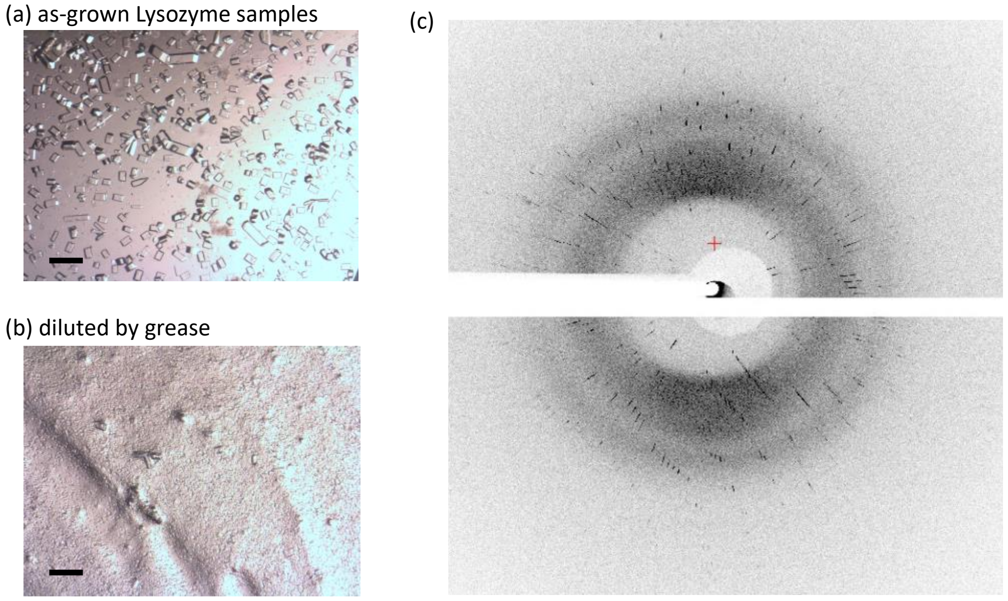

Supplementary Figure S12: Picture of lysozyme crystals (a) as-grown, and (b) after mixing with grease for diluting the crystal density to less than (100 μm)$^{-2}$. Scale bars are 100 μm. (c) An example X-ray diffraction image of the lysozyme microcrystals measured by ALD. The sample was diluted and loaded into T3. measured at BL40XU, SPring-8, using a large bandwidth high photon flux beam.

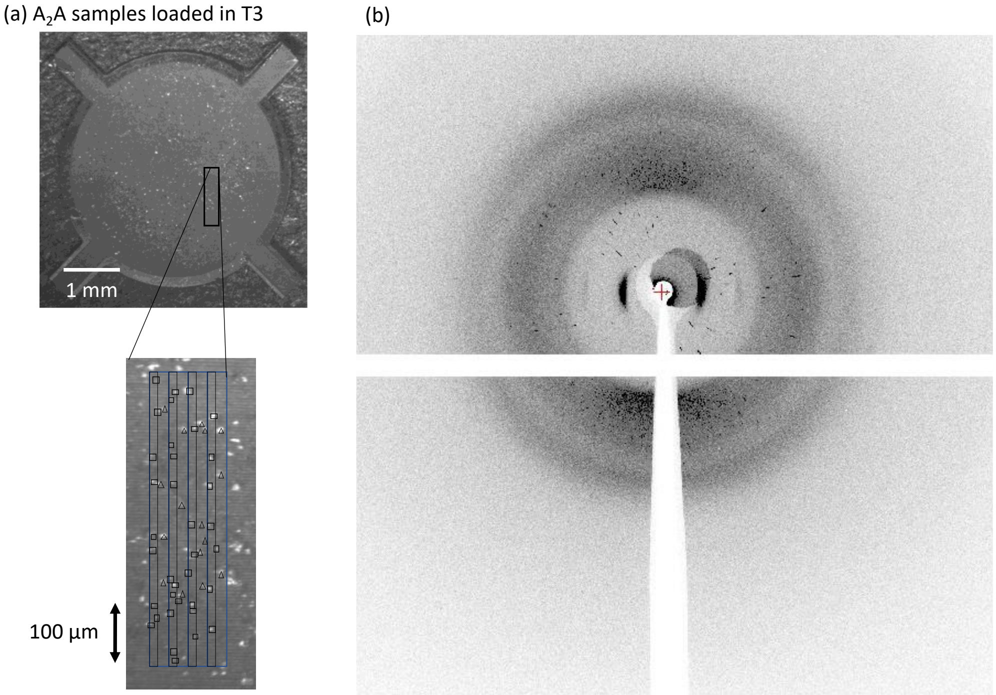

Supplementary Figure S13: (a) A_2A microcrystals with the size of 20-30 µm with the average spacing of ~100 µm grown in LCP. The top panel shows the picture of the sample loaded into a thin film sandwich sample holder T3. The lower panel shows the analysis of the sample efficiency (see main text). (b) An example X-ray diffraction image of the A_2A microcrystals measured by ALD using the sample holder S3.

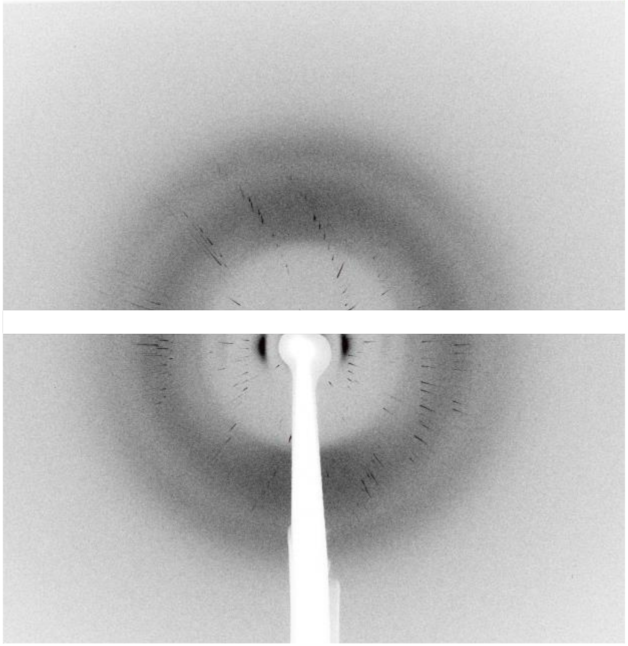

Supplementary Figure S14: An example X-ray diffraction image of KR2 microcrystals measured by ALD using the sample holder T3, with the sandwich of a pair of 25 µm-thick polyimide thin films fabricated by polyimide foil via laser cutting and ablation. The background scattering by the fibrous structure of the polyimide is visible as in the cases of Supplementary Figures S5, S6. The lack of the structured background in the experiment shown in Fig. 11 using T5 with the sandwich of a pair of 50 µm polyimide thin films fabricated by photosensitive resin is apparent.

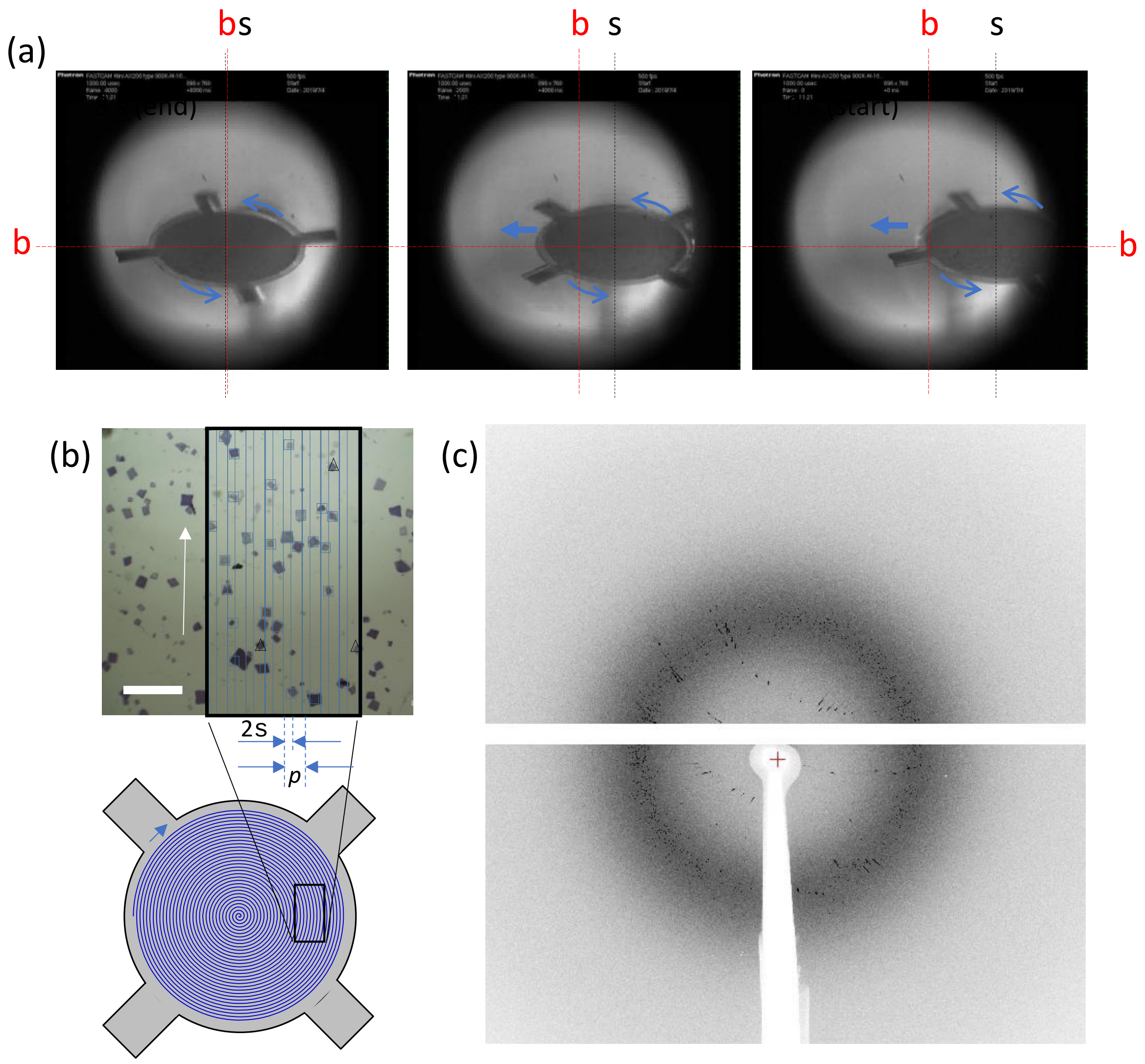

Supplementary Figure S15: (a) Snapshots of the thin film sandwich sample holder T5 containing LCP-grown KR2 microcrystals during data collection. From right to left, at the start (0 s), 4 s, and 8 s (at the end). The X-ray beam spot on the sample holder surface (marked as the intersection of the vertical and horizontal red chain lines) traces a spiral trajectory (sketched in (b)), in this case by the combination of sample rotation at 4 rps with transverse translation at 0.25 mm/s. The translation of the center of the sample holder, marked by the intersection of the vertical black line and the horizontal red line, with the progress of the data collection can be seen. (b) Picture of KR2 microcrystals with sizes in the range of 20 µm. The scale bar is 100 µm. The spiral sketches the X-ray beam trajectory for 4 rps rotation and 0.25 mm/s translation, with the separation between the neighboring trajectories of 62 µm. The fraction of the crystals that overlapped with the filled semitransparent rectangles with a width of 13 µm (corresponding to the beam spot size) was estimated to be 88%. (c) A typical diffraction image of the KR2 microcrystals acquired by ALD on the BL40XU beamline at SPring-8 using a large-bandwidth high photon flux beam using T5.

# refs	Compl (%)	Meas	SNR	d (Å)	R_{split} (%)	$CC_{1/2}$
941	100	780643	28.86	8.17	4.59	0.9906
858	100	479498	29.62	3.81	4.07	0.9942
846	100	394119	24.95	3.19	4.2	0.995
829	100	304036	18.32	2.84	5.46	0.9929
800	96.15	205076	13.87	2.61	7.23	0.9858
618	74.55	111177	10.06	2.44	10.26	0.9772
464	56.93	63293	6.87	2.31	15.05	0.9497
341	41.53	33772	4.55	2.2	19.4	0.9381
235	29.41	15738	3.35	2.11	31.39	0.8588
163	19.69	5957	1.71	2.04	78.11	0.6227

Supplementary Table S1: Statistics of the dataset of single-crystal lysozyme sample measured by ALD as shown in Figure 7.

# refs	Compl (%)	Meas	SNR	d (Å)	R_{split} (%)	$CC_{1/2}$
3672	100	460933	10.62	9.52	10.16	0.9817
3665	100	300856	7.88	4.39	11.95	0.9839
3627	100	247866	4.21	3.67	24.23	0.9538
3677	100	220402	2.1	3.27	52.49	0.8403
3671	100	194120	1.21	3.01	99.15	0.6545
3629	100	162833	0.82	2.81	160.89	0.4660
3653	100	129925	0.5	2.66	254.5	0.3438
3630	99.94	94734	0.27	2.53	457.53	0.2096
3677	99.54	64550	0.09	2.43	846.64	0.0832
3546	97.23	43520	-0.02	2.34	1289.35	0.1106

Supplementary Table S2: Statistics of the dataset of single-crystal KR2 samples measured by ALD: 10 datasets with 20,000 images were combined. From this dataset, the crystal structure was solved and shown in Figure 8(b).
