# Supplementary material for: Acoustic levitation and rotation of thin films and their application for room temperature protein crystallography
Source: Sci Rep. 2022 Mar 30;12:5349. doi: 10.1038/s41598-022-09167-z (PMC8967846; doi:10.1038/s41598-022-09167-z)
Supplement: Supplementary file 2 — Supplementary Information 2. [file 41598_2022_9167_MOESM2_ESM.pdf]

Supplementary information

Acoustic levitation and rotation of thin films and their application for room temperature protein crystallography

Michal. W. Kepa[1], Takashi Tomizaki[2, *], Yohei Sato[3], Dmitry Ozerov[2], Hiroshi Sekiguchi[4], Nobuhiro Yasuda[4], Koki Aoyama[4], Petr Skopintsev[1], Jörg Standfuss[1], Robert Cheng[5], Michael Hennig[5] & Soichiro Tsujino[1, **]

[1] *Division of Biology and Chemistry, Paul Scherrer Institut, 5232 Villigen-PSI, Switzerland*

[2] *Photon Science Division, Paul Scherrer Institut, 5232 Villigen-PSI, Switzerland*

[3] *Nuclear Energy and Safety Research Division, Paul Scherrer Institut, 5232 Villigen-PSI, Switzerland*

[4] *Japan Synchrotron Radiation Research Institute, Kouto 1-1-1, Sayo-cho, Sayo-gun, 679-5198 Hyogo, Japan*

[5] *leadXpro AG, PARK InnovAARE, CH-5234 Villigen-PSI, Switzerland*

* Corresponding author: takashi.tomizaki@psi.ch
** Corresponding author: soichiro.tsujino@psi.ch

Movie 1.mp4: high-speed-camera recording of rotating thin film sample T1.

Movie 2.mp4: high-speed-camera recording of rotating thin film sample T2.

Movie 3.mp4: high-speed-camera recording of rotating thin film sample T3.

Movie 4.mp4: comparison of rotation characteristics of disc-shaped film sandwich T3 with (left) and without (right) short blades.

Movie 5.mp4: reproducible angular position of levitated film sandwich sample T3 for pressure below rotation threshold.

Movie 6.mp4: visualization of air flow around a rotating thin film sample T1.

Supplementary Material:

- Description of the X-ray diffraction experiments and beamline optics at BL40XU, SPring-8,
- Evaluation of the average sample spacing from the observed hit rate for the X-ray diffraction experiments using the sandwich sample holder for dataset shown in Supplementary Figure S15.
- Supplementary Figures S1-S15 and Supplementary Tables S1-S2.
